# Life-Cycle Assessment in the Polymeric Sector: A Comprehensive Review of Application Experiences on the Italian Scale

**DOI:** 10.3390/polym12061212

**Published:** 2020-05-26

**Authors:** Ignazio Blanco, Carlo Ingrao, Valentina Siracusa

**Affiliations:** 1 Department of Civil Engineering and Architecture, University of Catania an UdR-Catania Consorzio INSTM, Viale Andrea Doria 6, 95125 Catania, Italy; 2 Department of Chemical Sciences, University of Catania, Viale Andrea Doria 6, 95125 Catania, Italy; ing.carloingrao@gmail.com (C.I.); vsiracus@dmfci.unict.it (V.S.)

**Keywords:** polymers, life cycle assessment, polymeric materials, sustainability, plastic, food packaging, LCA

## Abstract

In recent years, a growing media campaign has demonized the use of plastic tout court, as solely responsible for environmental problems. Behind what is now vulgarly called plastic there are actually many applications and uses without which our daily life would be greatly penalized in the most common and routine actions. Our belief, in the role of researchers who have made polymers and their derivatives their main research object, is that sustainable use of polymeric materials is not only possible but is above all necessary. For this reason, in this review which is part of the Special Issue “State-of-the-Art Polymer Science and Technology in Italy”, we offer a rundown of life-cycle assessment (LCA) studies on polymers used in the most important production and commercial sectors carried out in the last few years by Italians researchers.

## 1. Introduction

‘Plastic’ is now accepted as a universal term encompassing the huge array of polymers that are currently available in the market and are characterized by widely diverse chemical composition, mechanical properties, manufacturing methods and raw material feedstock (e.g., petroleum vs. renewable resources) [1]. This is what has made it possible to utilize them in almost every human activity [2].

Plastics present chemico-physical, mechanical, optical, and biological properties that, being adjustable for a huge number of disparate tasks, have contributed significantly to the expanding utilization of those plastics, making them increasingly prevalent in daily life [2,3].

It is estimated that since 1950 8.3 billion tons of plastics have been manufactured worldwide [1]. In 2018, global plastics production almost reached 360 million tons, with Europe contributing about 17% of that amount. The vast majority of the polymers utilized in the plastic industry are fossil-derived, which means that they are produced from crude oil and other fuels like natural gas and coal [1,3]. Most of them fall into the following categories, as defined by the Plastics Industry Society: poly(ethylene terephthalate) (PET), high-density polyethylene (HDPE), poly(vinyl chloride) (PVC), low-density polyethylene (LDPE), polypropylene (PP), polystyrene (PS) and ‘others’ [1].

**Figure 1:**
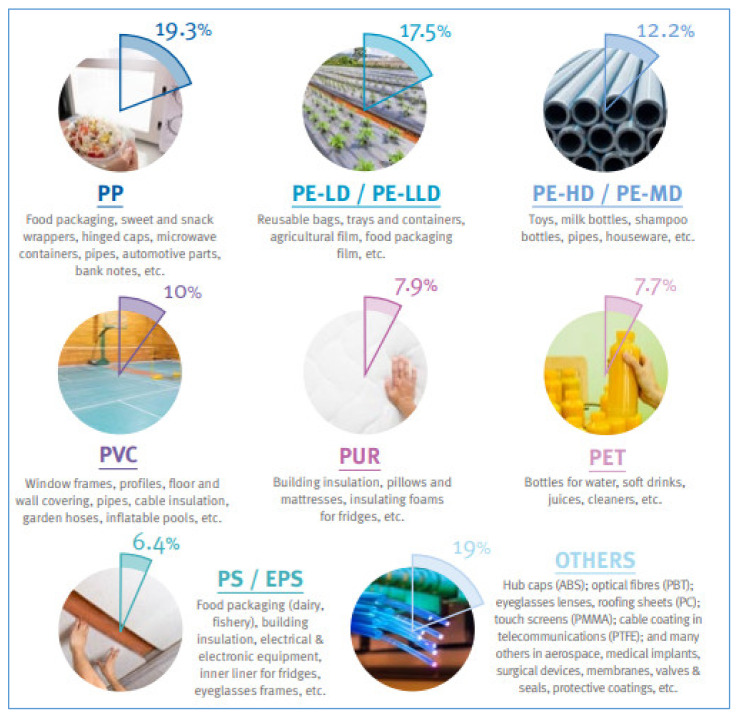
Distribution of plastics demand by resin types in the year 2018. Reprinted from [4], © 2020 with permission from PlasticsEurope.

Polyolefins, mainly PP and polyethylene (PE), are the leading polymers, covering around 50% of all possible applications [4], as shown in Figure 1.

The packaging sector is hugely demanding of those polyolefins and of other polymers, like PET whose core application is the packaging of water and other beverages [5], and PS for the production of fresh-food packaging trays [6]. The huge consumption of polymers in the packaging sector should be attributed to a combination of benefits including light weight, flexibility, strength, transparency, impermeability, and ease of sterilization [7,8]. 

Other highly demanding sectors are buildings, mainly because of the consumption of “hard” plastic materials like HDPE and PVC, and automotive, due to utilization of PP and other thermoplastics. Additionally, agriculture is a big consumer of PP and LDPE, which are utilized mostly for the production of protected cultivation films, nets, pipes and irrigation/drainage systems, and packaging [9]. The wide range of these applications have led to the elaboration of the new term ‘plasticulture’, to strengthen the importance that plastic commodities and related manufacturing industries have in agriculture [2,10].

All over Europe, consumption of plastic films for covering tunnels in A-shaped greenhouses and in tunnel-greenhouses is equal to an average of 72,000 and 75,000 tons per year, respectively [11,12]. A large part of this consumption comes from the Mediterranean region, especially from those countries like Italy where protected crops are largely cultivated [12]. In Italy, agricultural plastic consumption was estimated to be 350,000–400,000 tons per year, with 30% in the field of greenhouse claddings, low tunnels, soil mulching, vineyards nets and so on [11,13].

**Figure 2:**
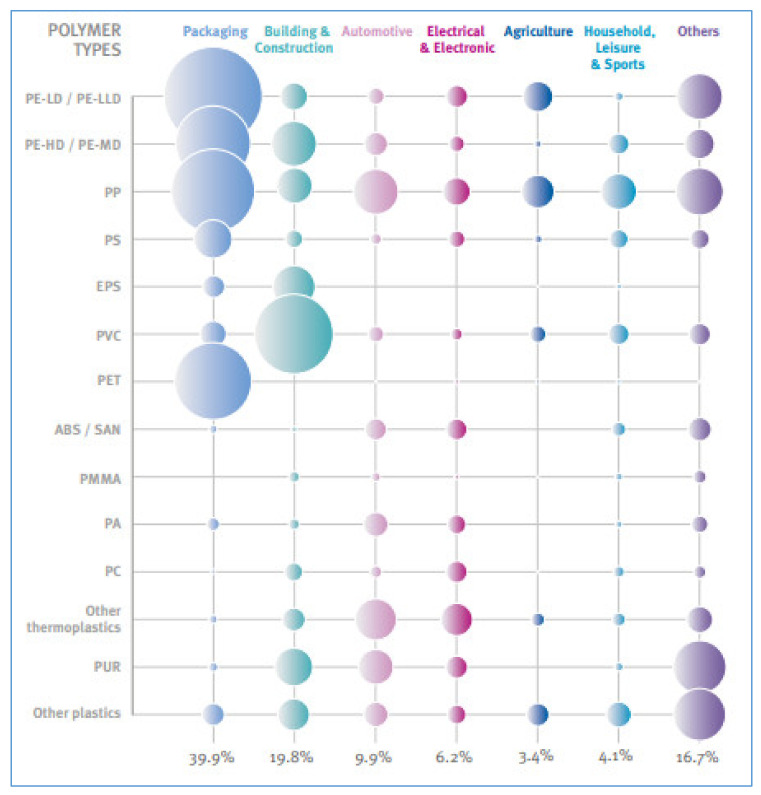
Plastics demand by segments and polymer types in 2018. Reprinted from [4], © 2020 with permission from PlasticsEurope.

That said, current plastics-demanding sectors are depicted in Figure 2, as extrapolated from the latest PlasticsEurope’s report [4].

The environmental impacts caused by the plastic industry are increasingly being understood, and so are increasingly gaining the attention of scientists, producers, company managers and owners, as well as policy- and decision-makers, at the international level [1,14]. 

To perform the functions that they are designed for, plastics cause a set of environmental impacts, in almost all phases of their life cycles, mainly deriving from exploitation of primary-energy resources and from the emission of greenhouse gases (GHGs) and of other polluting substances [6,15]. Furthermore, the massive consumption of those products results in consistent waste generation, with environmental and socio-economic consequences harmful to the heath of humans, animals, and plants and to the quality of the global ecosystem [16]. Parlato et al. reported that 26 million tons of plastic waste get generated every year in Europe and that just 30% of those is recycled, so representing a great loss from an economic and environmental viewpoint [17]. Still, the environmental benefits of recycling are well documented, with lots of studies available in the literature. Recently, in the field of protected crop cultivation, Cascone et al. [12] explored relevant energy and environmental issues associated with the recycling of post-use greenhouse-covering film into LDPE granules usable, as an intermediate product, in a wide range of downstream systems. The study highlighted that recycling is highly energy and water demanding, as well as highly GHG emitting. Despite this, the delivered (recycled) LDPE granulate was far more sustainable than—and so was validated as a feasible substitute for—the virgin counterpart.

Plastics are mainly produced for durable scopes, which makes them able to persist undegraded for decades in the environment where they are disposed of, with a persistence time going well beyond the predictable one [3,16,18,19,20,21]. It is this persistence making plastics accumulate in increasing quantities in the marine environment [22]. This should be attributed to marine-water bodies, like oceans and seas, being downhill and downstream virtually from every human living place, and to around half of the world population living within 50 miles (around 80 km) of those bodies. Consequently, lightweight plastic trash, like bags, bottles and cutlery [16], because of the lack of effective recovery systems, blows and runs off into the sea [22]. That is when it starts moving to a huge number of habitats, with a set of significantly damaging impacts to humans and to wildlife [22]. 

These and other related concerns emphasize the need to push towards sustainable, disposal scenarios that are oriented to recycling and so to the production of quality secondary raw materials [12]; and to alternatives to petroleum mineral-based products [16].

In this regard, attention is being increasingly focused upon the development of eco-friendly, innovative materials: chief among them, polymers derived from biological feedstocks, known as *biopolymers*, have been documented as effective to replace unsustainable, fossil-derived plastics [2,23].

They represent, in fact, a renewable substitute to the traditional petroleum-based plastics, and can be derived from different biomass types, including agricultural products, such as corn or soybeans, as well as algae or food waste [2]. Both recycled-polymers and biopolymers can contribute to greening the plastics sector, and accelerating the transition towards sustainable, equitable, post-fossil carbon societies [24].

The increasing utilization of polymers in the multitude of sectors shown in Figure 2 is one good reason for development of environmental assessments as the essential base to find the improvements that can be made in the life cycle of plastic products. Life-cycle assessment (LCA) is one of the tools that are currently available in the market and can be used for such a purpose. Walker and Rothman recommended its use to compare the environmental impact of bio-based and fossil-based polymers and, so, to account for the important balances and trade-offs between polymers and between impacts, which is essential in order to make sensible material selection [1].

LCA of products and processes is widely recognized, with two main beneficial features that are related to the ‘cradle-to grave’ approach, and to the use of a functional unit (FU) that enables comparative evaluations [25]. LCA is based upon a clearly structured methodology that is ruled by International Standards 14040 and 14044 [26,27]. It is a systematic tool that enables qualifying and quantifying the relevant environmental loads and impacts that are associated with the life cycle of a product or service [6,25]. Preparation of raw and auxiliary materials from resource extraction, transportation, manufacture of the product, end use and ultimately disposal of the product itself are accounted for in LCAs [25,28]. 

LCA has been significantly improved over the past decades, mainly thanks to the development of activities for: improvement of currently used databases; integration of quality assurance; improvement of completeness, transparency and consistency of assessments; and harmonization of methodological aspects and applications [25,29,30]. This has contributed to its emergence as a valuable decision-support tool for both policy makers and company owners in assessing the cradle-to-grave impacts of a product or process. 

Over the years, practitioners worldwide have used LCA to explore relevant environmental aspects in a wide range of sectors including automotive, buildings and construction, energy, electronics, agriculture, and food production and packaging [6,11,31].

**Figure 3:**
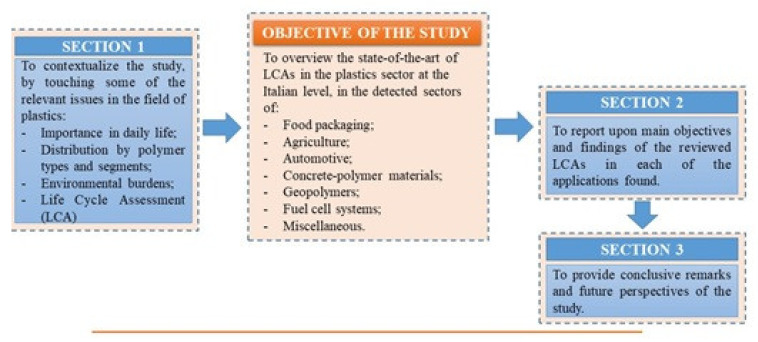
Review’s flowchart.

In this context, this study was aimed at overviewing the state-of-the-art of LCAs in the plastics sector at the Italian level. This has been done to complement the Special Issue about the “State-of-the-Art of Polymer Science and Technology in Italy”, by providing information on the relevant environmental issues of plastic material applications in the Italian region (Figure 3). 

The study was motivated by the importance of making polymer supply chain actors aware that a sustainable use of polymeric materials is not only possible but is above all necessary. In this way, there is greening not only of life cycles of those materials but also of the downstream systems in which they are utilized as such and/or are processed into finished, more complex commodities. This review offers a rundown of LCAs on polymers used in the most important production and commercial sectors, carried out in the last few years by Italian researchers.

## 2. Applications

The authors of this paper reviewed the findings of relevant articles exploring the application of LCA in the polymeric sector. The bibliographical search was conducted by browsing scientifically-recognized databases, like ‘Scopus’, ‘Web of Science’, ‘Science Direct’, and ‘Springer Link’. Combinations of key-words connected with the research field investigated were used by the authors, and included: ‘polymers’, ‘plastics’, ‘environmental sustainability’, ‘carbon footprint’, and ‘life-cycle assessment’. To be consistent with the objectives of this study, papers conducted at the Italian level were then selected and were extrapolated from the ensemble of papers found. A total of 58 LCAs were detected by the authors in several research areas, including food packaging, agriculture, automotive, admixed concretes, geopolymers, and fuel cells. Therefore, it can be highlighted that Italian research touched on most of the applications shown in Figure 2. The biggest number of LCAs (19) were found in the food-packaging sector, and regarded films, trays and bottles.

Finally, many of the reviewed papers were authored by team members belonging to different universities and research institutions, so remarking that environmental research on polymers presents such a complexity that it requires investigation through a multidisciplinary approach.

Each of the papers are discussed in the following sections in terms of key objectives and findings: additional information may be gathered by the reader from the original papers.

### 2.1. Food Packaging

**Figure 4:**
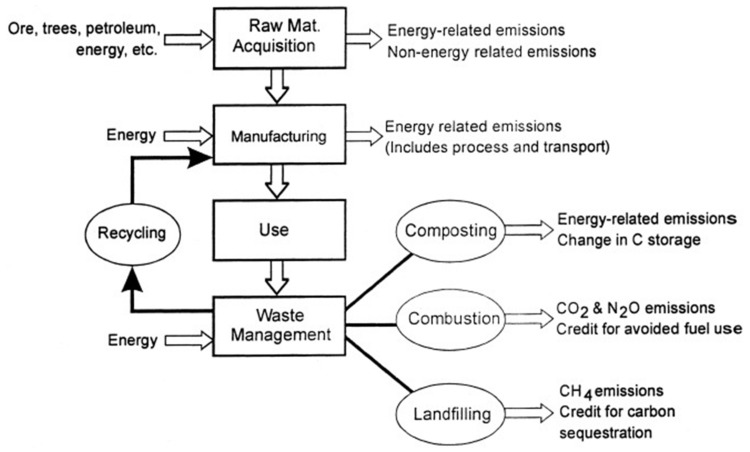
Municipal solid-waste life cycle. Reprinted from [36], © 2020 with permission from Wiley.

**Figure 5:**
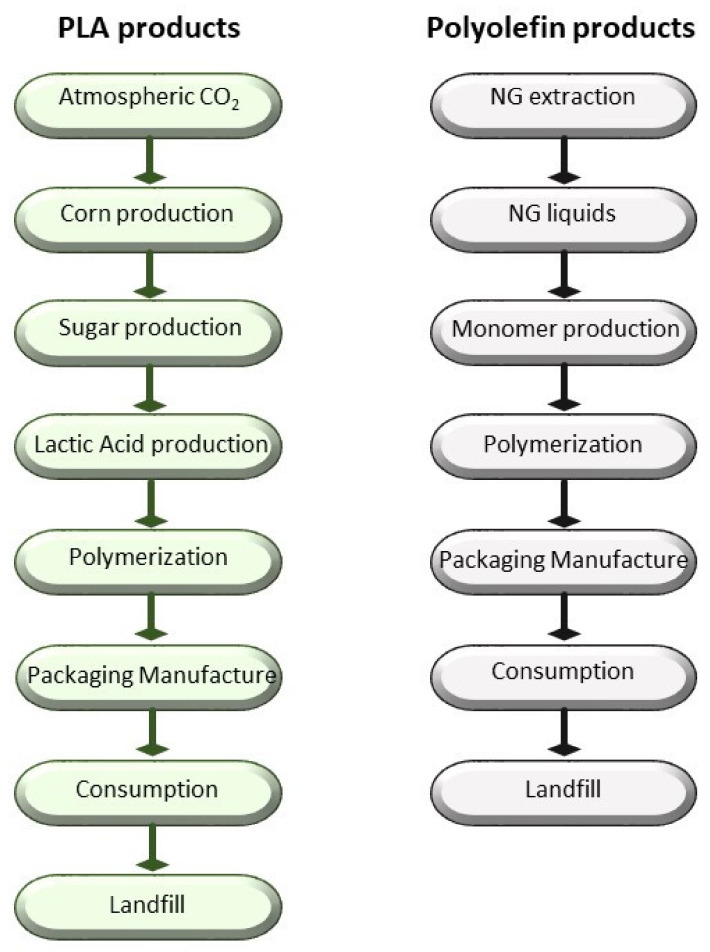
System boundaries for biodegradable poly(lactic acid) (PLA) and synthetic polyolefins.

As reported by Siracusa et al. [32] and as can be extensively read on the European Bioplastics website [33], the largest field of application of extracted crude oil is for plastics, with a current global production of 200 million tons and an annual grow of about 5%, with a consequent concern about ecological problems due to their total non-biodegradability [34]. As mentioned in the Introduction, within the petrochemical-based plastics materials, PET, PVC, PE, PP, PS and polyamide (PA) are the most common and used in the food-packaging field because their large availability, low cost and good performance such as tensile and tear mechanical strength; being a good barrier to oxygen, carbon dioxide and aroma compounds; heat sealability; and so on. Considering that when they are used as food-packaging materials they are not totally recyclable, also because they are often contaminated by foodstuff and biological substance, several thousands of tons of goods are every year landfilled, increasing the problem of municipal waste disposal [35]. The growing environmental interest imposes on packaging polymers as well as the technological process both user-friendly and eco-friendly attributes. In order to take into consideration this concern, LCA is a useful technique to evaluate the environmental impact of such materials, considering two fields of interest. The first one is the final destination of post-consumer plastics, comparing recycling with other options such as source reduction, incineration and landfilling as well as energy conversion and chemical recycling options. The second one is to take into consideration alternative materials such standard glass and/or metal packaging and/or biodegradable polymers that could be recycled or composted. These options must be considered in their entire life cycle and can be summarized in Figure 4, using the usual scheme of the LCA methodology:

As reported before, for many years, polymers from fossil fuel resources have supplied most common packaging materials such as glass and steel, thanks to their great features especially softness, lightness and transparency. Due to their non-biodegradability, plastics use has led to serious ecological problems. Despite their total substitution with eco-friendly materials being just impossible to achieve in a near future, especially for specific applications such as food packaging, the use of bioplastics (biodegradable and/or compostable materials) should be considered. In this context, biodegradable polymers as well as bio-based polymers are being promoted as a good environmental alternative to the conventional plastics, derived from petroleum resources. They can be obtained from renewable resources or from waste, as food and plants, and can be recycled [32]. If landfilled, they become carbon dioxide and water, through composting. The main biodegradable polymer, compared with the conventional ones, is poly(lactic acid) (PLA), whose properties and performance make it suitable for food packaging application but not with success in every field, such as for example not for carbonate beverage bottles. LCA is a good tool that could be used to assess the overall environmental burden of a product and of the system used for manufacturing, use and disposal of such product. System boundaries have to be perfectly defined and are needed in order to clearly separate the system from the environment. As a basis for this analysis, the system boundaries for a biodegradable polymer such as PLA and conventional plastics such as PP, PE, PET, PS and so on, are illustrated in Figure 5, as an example of the most common polymers employed for packaging application.

After use, package is discarded and, most of the time, is landfilled. In many industrialized countries other treatments could be considered and are used, with important implications for the LCA results. As reported by Costa, Quintero and Dias [37], until December 2018, 144 articles written in English appeared in international literature dealing with the LCA application in the field of plastics materials; of those, 49 articles were devoted to real case studies and the remaining devoted to reviews, viewpoint, mixed approaches and methodological development articles, presenting the LCA foundations, main methods, current operation state and challenges. Several articles have focused their attention on energy consumption, global warming potential (GWP), LCA of mechanical recycling technologies and on processes. The major numbers of articles are from Germany (22), followed by the United States of America (16), Italy (14) and China (13) and then The Netherlands (10), Spain (6) and France (5). While the USA and Germany reported each one 11 articles on real LCA cases study, Germany and Italy reported each one 11 contributions as reviews and viewpoints on the development of the LCA methodology. Choosing Italy as geographically representative situation, in the knowledge of the authors until 2020, the manuscripts dedicated to the food-packaging plastics materials total 20, discussed in this review, in order to understand the use of such methodology in this important field of interest. 

Mainly, two kind of plastic materials were the object of such LCA studies: materials obtained from non-renewable fossil fuel resources, as mentioned in the manuscript, and biodegradable polymers, obtained from renewable resources, such as PLA. It must be considered that also other materials are fully used in the foods packaging field, such as mixed materials, multilayer films, bio-based materials and polymers derived from microorganisms such as polyhydroxyalkanoates (PHAs), but in lower amounts due to the high price and lower performance.

Among the series of synthetic polymers present in the plastic market and especially in the food-packaging field, one of the most used is PET, with a global production of 30.3 × 10^6^ tons in 2017 [38], and consequently the most present one in plastics urban waste, followed by PE and PP. Its performance, characteristics and environmental impact were compared with biodegradable polymers coming from renewable resources. Among the biodegradable polymers, PLA is surely the most used in the packaging field and studied in its technological characteristics and performances. One of the first manuscript dealing with a LCA study on such a polymer, but not in Italy, was that of Bohlmann [36], who carried out a LCA comparing PLA and PP, used as food packaging materials. In this first work, four energy main categories were considered, including fuel production (e.g., electricity consumption), fuel use for processing (e.g., steam consumption), transportation energy and feedstock energy. The selected functional unit was one metric ton (1000 kg) of yogurt packed in plastics, purchased by the consumer. In order to permit comparison with other LCA manuscripts, the energy consumption per kg of polymer used as packaging materials was also considered. Stanford Research Institute (SRI) Consulting’s Process Economics Program (PEP) data were used, as good source of information for mass and energy balances. It was assumed that filling, distribution and use of PLA and PP yogurt cups was exactly the same. A cradle-to-grave analysis was performed, focusing on greenhouse gas emissions as the most relevant impact category due to the fact that for SETAC (Society of Environmental Toxicology and Chemistry) it is the only indicator with great relevance for biodegradable polymers such as PLA. Consequently, emissions of greenhouse gases are converted to metric tons of carbon equivalent (MTCE) and normalized for kg of polymer and for one ton of packed yogurt FU. As reported by Bohlmann, due to the fact that PLA consumes almost no feedstock energy, this polymer is more energy efficient than PP for food packaging application. Furthermore, PLA and PP greenhouse gas emissions become equivalent if it is considered that the carbon embodied in PLA is fully landfilled. Considering that the biodegradation of PLA in landfill could have an important effect on greenhouse gas emissions, uncertainties could be present in the LCA results. In addition, data analysis can be affected by uncertainty due to the fact that many plants in the world produce PP with differences in processes, energy usage and environmental loads from plant to plant. For PLA instead, there was only one commercial plant [39] thus making unavailable a sufficient number of data. To make the comparison of data the most consistent possible, Bohlmann chose the PEP database. Subsequently, several authors applied the LCA methodology on plastic materials. In this review we have focused our attention on Italian case studies.

In 2010, Girone and Piemonte [40] analyzed two different disposal scenarios, such as composting and recycling processes, for PLA (made from Nature Works) and Mater-Bi (made from Novamont) considering the land-use change (LUC) emissions, comparing also cost and benefits of the bioplastic disposal versus conventional plastics. In 2011, the same authors [41] realized a cradle-to-grave LCA study of PET compared with PLA bottles for drinking water, assuming the same waste scenarios like landfill and incineration. The results obtained highlighted that, despite the benefits related to the use of renewable resources for the production of biodegradable bottles in respect of non-renewable resources for PET production, higher impact value for the categories human health and ecosystem quality were recorded, due to the use of pesticides, consumption of land and water for the production of row PLA. However, it must be considered that LCAs’ unfavorable results were related to the fact that in 2011 the technology for PET production and recycling was much more mature, tested and optimized both for production and recycling processes in respect to that of PLA, which was at an early stage of development, with a very limited and localized production. The results were interesting when different final dispositions were compared: recycling for PET and composting and recycling, in different percentages, for PLA. Authors reported that the best benefit in the use of PLA could be achieved only if it could be totally recycled because composting and incineration showed a higher environmental impact than recycling. Quite similar results have been obtained when recycling of 100% PET bottles was compared with incineration/recycling, in the amount of at least 20%–80% of PLA bottles. The same mixed scenarios as final bottles destinations have been also considered. In particular for PET 70% recycling and 30% landfilling, 70% recycling and 30% incineration and finally 50% recycling, 30% incineration and 20% landfilling; for PLA 50% recycling and 50% landfill, 50% recycling and 50% composting, 40% recycling 30% composting, 15% landfilling, and 15% incineration. Comparable results were obtained when the macro category “resources” was considered. 

In the same year, 2011, Piemonte [42] carried out an LCA cradle-to-grave analysis comparing the results obtained choosing as functional units 1000 shells of PLA, polymer produced from Nature Works (USA), and 1000 shells of Mater-Bi, a starch based bioplastics, made from Novamont (Italy), both used as food packaging materials. The scope of their work was to assess the energy and GHGs emissions coming from the production of bioplastics, compared with the standard materials, as well as the best waste final disposition, in order to maximize energy saving. The Intergovernmental Panel on Climate Change (IPCC) and Global Warming and Cumulative Energy Demand (CED) methods were used, with an end point approach, considering three ‘‘Damage’’ categories such as human health, the ecosystem quality and the non-renewable resources depletion as the most high-impact categories. PET and PE data, used for comparison, were based on the Eco-profiles of the European plastics industry. Piemonte found that the replacement of the petroleum-based plastics with bioplastics could be considered an interesting strategy towards the sustainable development, for energy and GHG emissions savings. As a final destination of bioplastics, mechanical recycling could be considered a good alternative, maximizing the energy saving and reducing the renewable resources consumption. In addition, Piemonte underlined that the results reported are interesting in terms of the environmental reliability of bioplastics, but cannot be taken as guidelines for the market use of PET or PE rather than PLA or Mater-Bi, and vice versa.

In 2013, Toniolo et al. [43] analyzed with the LCA methodology two kinds of plastic product: a multilayer plastic tray and a PET tray, used for food packaging. For the first product an end-of-life scenario that includes land-filling and incineration was chosen, whilst for the second product an end-of-life scenario comprising recycling, land-filling and incineration was considered. They studied how an innovative and recyclable package material could be environmentally preferable in respect to a non-recyclable one, explaining how the utilization of recycled materials represents a great effort to reduce the environmental burdens. The package produced employing a recyclable mono-material film is more environmentally advisable than the multilayer ones, for all of the impact categories analyzed. The results were also tested by a sensitivity analysis and an uncertainty analysis in order to confirm the results of the life-cycle impact assessment. The study was used to demonstrate that the life-cycle approach is an important tool to assess how a prevention activity to reduce waste production is actually an environmentally sustainable alternative, providing also great decision-making support in the field of packaging waste management. In 2017 they completed their research considering allocation strategies for implementation in the modeling of the burdens of input waste and end-of-life disposal of such food-packaging materials [44]. 

During 2014, three papers appeared on LCA studies: one dealing with an environmental assessment study of a multilayer material bag used for food packaging [45], the second one dealing with an economic and environmental assessment study performed on reusable plastic containers used for a food catering supply chain [46], and the third one based on the environmental assessment of two packaging materials for poultry products, such as a polystyrene-based tray and an aluminum-based trays (70 wt% primary and 30 wt% secondary aluminum) [47].

In the first paper, Siracusa et al. reported an LCA case study on a multilayer film bag made of PA and LDPE, used for vacuum or modified atmosphere packaging (MAP) technology food preservation. Following the ISO standards 14040:2006 and 14044:2006 rules, a functional unit of 1 m^2^ of plastic film was chosen, with a cradle to factory-gate approach, including the phases of the raw materials for bag production and processing and the bag’s delivery to both the food production and packaging plant. Due to the use of crude oil and natural gas, the most impacting phases were the production of PA and LDPE granules. The most affected damage category was “Resources”, followed by “Climate Change”, “Human Health”, and “Ecosystem Quality” respectively. Film thickness reduction and the use of recycled PA granules were proposed to the firm in order to reduce the total damage. The assessment results showed that the two proposals allowed a reduction of about 25% and 15% (respectively) of the assessed damage.

In the second paper, Accorsi et al. reported that during the last few decades, Europeans have drastically modified their food consumption habits through the food catering sector, and chosen to eat out or buy take-away foods, thus increasing drastically the amounts of package waste, exacerbated as the food catering chain service does not currently employ reusable packaging systems. They compared a multi-use system to traditional single-use packaging (e.g., wooden boxes, disposable plastic crates and cardboard boxes), used for a fresh fruit and vegetable Italian catering chain, from vendors to final customers, to quantify the economic returns and environmental impacts of the reusable plastic container (RPC). A carbon footprint (CF) analysis was performed for the environmental assessment. As plastic materials, 1 kg of PP for the production of 1 kg of single-use plastic crates and RPCs was considered, while if an amount of 2.8% of scrap is added during the production phase, 0.925 kg of polypropylene granulate can be expected to yield one kilogram of product. The LCA results demonstrated that the environmental impact associated with the single-use network was mainly due to the manufacturing phase, due to the great volume of the packages required over the year. It was observed that the transportation phase, as well as the different disposal scenarios, significantly affected the sustainability and the environmental impacts of the RPC system. From the study it was observed that the RPC system will lead to a reduced environmental impact in terms of CO_2eq_ emissions while the overall economic return, in the opinion of the authors, is projected to be negative, due to a cost increasing of about 0.06 € per kilogram of handled food product. 

In the third paper, Zampori and Dotelli described a cradle-to-grave case study, considering the production, use phase (such as cooking), and different end-of-life scenarios. Greenhouse Gas Protocol (GGP), Cumulative Energy Demand and International Reference Life-Cycle Data System (ILCD) midpoint method were used for the life-cycle impact assessment (LCIA) measurements. The aluminum bowl was designed for use during the cooking stage of the poultry product and to reduce the cooking time allowing consequently a reduction in electric energy equal to 0.21 kWh (1.38 kWh instead of 1.59 kWh), that is of primary importance to reduce overall emissions, expressed as CO_2eq_ emissions, especially in those countries, such as Italy, where the use of fossil fuels is predominant for electric energy production. As regards the production stage of the two trays, it was found that the PS tray was more sustainable than the aluminum one. Furthermore, in order to reduce the environmental impact, the trays were designed to lower the overall emissions, determined by GGP and CED. In particular, it was found that the Al tray was less sustainable than the PS one, according to a “recycled content” scenario, but it was more sustainable when a “substitution” scenario was applied. In particular, the authors found that if an Al tray made of totally recycled material is used, also if this is less sustainable than a PS one, the choice of the Al tray becomes the most sustainable option thanks to the lower impacts recorded during the cooking stage that were calculated to be the most impactful over the entire life cycle of the two alternatives by a “cradle-to-grave” approach. 

In 2015, Ingrao et al. [6] performed an LCA study of foamy polystyrene trays used for fresh meat packaging. The FU was identified with 1 kg of packed-trays delivered to food production and packaging firms, with a maximum capacity amounting to almost 800 cm^3^ of food. The tray size was chosen as the object of the study because it was the most commercialized one amongst the other different types produced by the Italian firm and so representing perfectly its core-business. The study highlighted that the highest environmental impacts come from PS-granule production and electricity consumption. The authors underlined as there were no margins for improvement in the production of PS granules, in the transport of materials and of the trays’ inputs. On the contrary, changing the energy source into a renewable one (by installing, for instance, a wind power plant) would enable a 14% damage reduction. 

In 2015, Conte et al. [48] focused their attention on 24 scenarios of packaging used for cheese obtained from sheep. The goal of the study was to analyze the environmental implications of different packaging systems, considering the potential food loss, in order to obtain an eco-indicator able to quantify the environmental effect of different packaging materials. The geographical context was referred to two area of the Basilicata region: Matera and Potenza (South of Italy). The LCA methodology was applied following two approaches: an attributional approach (AA), in order to consider the impacts related to the adoption of different packaging solutions, by taking into account only the phase of packaging and including the production and disposal phases of packaging materials; and a consequential approach (CA), with the aim of obtaining information about the consequences of the decisions in different packaging choices. In this last case, the investigation was oriented to the environmental implications in using different plastic films and gaseous atmospheres, together with the inside effects such as the production and disposal of plastic materials, and outside effects such as shelf-life and food losses. In this context, four types of plastic films were studied: a multi-layer high-barrier film of PET (13%) co-extruded with PE (78%), exposed to anti-fog treatment (9% AF); a common multi-layer film of polyamide (21% nylon), PE (74%) and ethylene vinyl acetate (EVA) (5%); oriented polypropylene (OPP) (100%) of 60 micron thickness; OPP (100%) of 35 micron thickness. For each heat-sealed bag, composed as described above, six gaseous atmospheres were considered: air, vacuum and four MAP obtained by mixing N_2_ and CO_2_ at various percentage. Two scenarios were considered for packaging disposal: the multilayer systems recovered for energy use (incineration) whereas the PP was completely recycled. The authors found that, if an attributional LCA approach was followed, thinner and recyclable packaging materials resulted in being more sustainable while if consequential type approach was followed, packaging able to guarantee a longer shelf life, thus reducing the food loss become the most sustainable. Furthermore, considering that the environmental impact caused by cheese production is generally high if it is compared with the other stages of the life cycle, the packaging possibility to reduce food losses was a key factor from an environmentally proper choice point of view, more than packaging production and/or packaging disposal.

Accorsi et al. [49], in 2015, reported a LCA case study on extra-virgin olive oil (EVOO) bottled in glass, PET and recyclable PET (R-PET), quantifying the impacts of global warming potential, ozone layer depletion, non-renewable energy use, acidification, eutrophication and photochemical smog. They focused their attention on the EVOO processing and bottling, on the EVOO and packaging supply, on the distribution to customers and on the management of end-of-life (EoL) treatments (recycling and disposal of wastes). The functional unit was a 1 L bottle of EVOO (i.e., equal to 0.916 kg·L), supplied by an Italian olive oil firm. The LCA analysis was performed according to an attributional and a consequential approach, in order to quantify the impact of the different technological approach. The authors found that if high fractions of package waste is devoted to recycling (over 40%), EVOO bottled in glass becomes less impactful than PET. Finally, LCA highlighted the potentiality of R-PET bottles in reducing the environmental impact of EVOO supply chains.

In 2017 three papers appeared on LCA case studies. The first one was performed on cheesecake normally packaged in low-density polyethylene film [50], with a limited shelf-life due to fast microbial growth. The authors decided to study the effect of different packaging conditions like use multilayer gas barrier trays, subsequently wrapped with a multilayer gas and water barrier film (i.e., AerPack packaging), in order to extend the food shelf life. As control batches, food was packaged in gas barrier recycled PET (named XrPet) trays and wrapped with XrPet films. Samples were characterized not only from a chemico-physical, microbiological and sensory parameters point of view, but an LCA study was also carried out, considering those new packaging solutions a possible way to minimize transport costs and to generate economies in manufacturing. The chosen FU was a unit of food sold, corresponding to a tray containing two cheesecakes, with a total weight of approximately 300 g. The ReCiPe endpoint method was used for this LCA study. Metal depletion, followed by human toxicity, freshwater and marine ecotoxicity were the impact categories that varied the most, relating to a high environmental impact of packaging. The ReCiPe normalized results confirmed that the AerPack materials showed a lower environmental impact than the XrPet. Moreover, considering the economic sustainability approach, the authors underlined as a change in the packaging technology to keep the product fresh longer, could allow the cheesecake producer to minimize transport costs, generating economies in manufacturing. By contrast with what was considered by Conte et al. [48] that provided an eco-indicator index able to quantify the environmental indirect effects related to the different choices in the food packaging, Gutierrez et al. [50] utilized an economic model considering the minimum amount of product delivered on consignment that must to be sold, defining the impact of shelf life on the food losses. 

**Figure 6:**
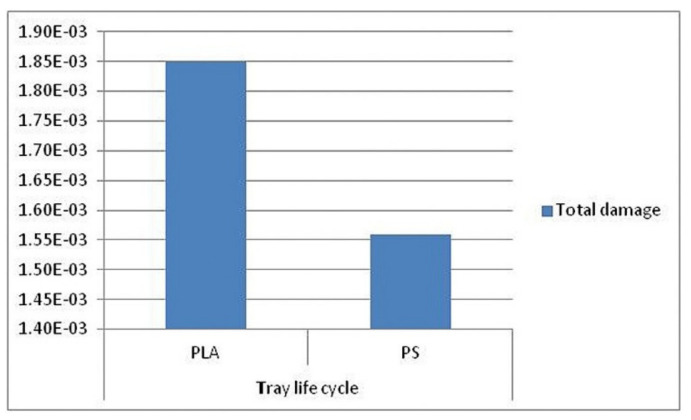
A comparison of the (total) damages associated with the life cycles of PLA and polystyrene (PS) trays. Values are expressed as pt.kg_tray_^−1^. Reprinted from [16], © 2020 with permission from Elsevier.

**Figure 7:**
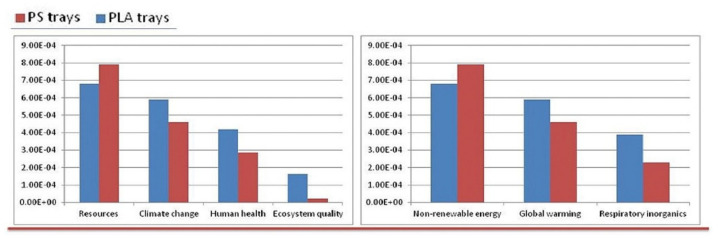
A comparison of the damages associated with both DCs and ICs in the life cycles of PLA and PS trays. Values are expressed as pt.kg_tray_^−1^. Reprinted from [16], © 2020 with permission from Elsevier.

The second paper, published by Ingrao et al. [16], reports the evaluation of life-cycle environmental impacts for fresh-food packaging trays constituted by expanded-PLA (EPLA), compared with standard trays used for fresh meat, constituted of expanded PS (EPS). The purpose was to understand if the biodegradable polymers could be an interesting and environmentally valid alternatives to the synthetic ones, by assessing the global environmental impact but considering more damage and impact categories (DCs and ICs) than those reported in previous studies by the same authors [3,6]. In particular, the comparison was carried out using an end-point approach level and the results were shown to be associated with the life-cycle of the trays (Figure 6) and the DCs and ICs (Figure 7). In both figures, values were expressed as pt kg^−1^.

The authors concluded that the most impacting phase were represented by the production of PLA granules, due to the corn-cultivation phase, by the energy consumed in the processing of those granules and, mostly, by their transportation from America to Italy, and in Italy direct to the tray manufacturing factory. In this context it was observed that the transport issue was responsible for the PLA-trays’ higher impacts, compared with the PS ones.

The third paper was reported by Cinelli et al. [51], evaluating the degradability in compost of some representative examples of materials prepared with pristine PLA, PLA with chitin nano-fibrils (PLA/NC), PLA with NC and polyethylene glycol 400 (PLA/NC/PEG400) used as plasticizing additive, suitable to be used for production of rigid or flexible packaging. Due to the fact that a biodegradable polymer was blended with an active compound that might hinder the compostability of such materials, for authors it was important to assess the test of degradability on those innovative materials, bio-based and containing biodegradable components. The functional units were respectively 1 Kg of nano chitin fibrils, and 1 Kg of PLA/NC/PEG400 pellets. The materials based on PLA and NC showed disintegration in compost, evidencing no toxicity, while the production process of NC was affected by a high consumption of water and chemicals. LCA analysis measured the environmental benefits for PLA/NC/PEG400 products, mainly related to the renewable sources of most of the used components such as chitin, chitosan, PLA, glycerol, as well as the compostability of the obtained product. The LCA study was aimed at suggesting improvements for the production of NC based materials in terms of sustainability and future application in food packaging market. 

A year later, Petrucci et al. [52] reported a comparison between limonene plasticized PLA films containing cellulose nanocrystals (CNC) and an acetyl tributyl citrate (ATBC) plasticized PLA films, having equivalent mechanical properties, containing organo-modified montmorillonite (OMMT). A cradle-to-gate approach was followed, using the Eco-Indicator 99 as the main method to assess the environmental impacts and 1 kg of plastic film as the functional unit in order to identify the most critical inputs. Physical and geographical limits of the investigated PLA whole life cycle, including both nanocomposite manufacturing plant and the composting end of life, were considered as system boundaries. The film service life and transports were excluded from the study. The authors found that the chemical extraction of CNC caused a large environmental footprint and a relatively relevant energy expense, despite it being a biobased filler. Furthermore, the environmental impact of PLA/limonene film reinforced with 1% in weight of CNC (PLA/CNC/limonene) was comparable to the environmental impact of PLA films reinforced with OMMT and plasticized with a petroleum based plasticizer (ATBC) (PLA/OMTT/ATBC). 

**Figure 8:**
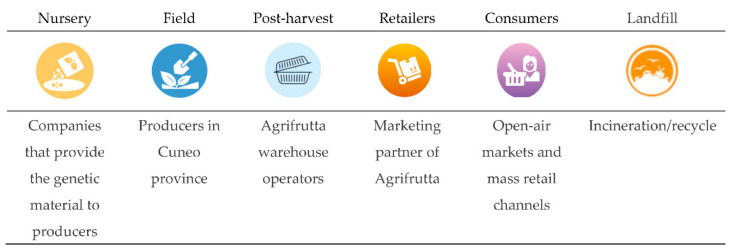
Raspberry supply chain framework. Reprinted from [53].

In 2019, Blanc et al. [53] performed an LCA case study of raspberry supply chains from an environmental, economic, and social point of view, using bio-based plastics packaging. LCA, life-cycle costing (LCC), and externality assessment (ExA) methodologies were applied to assess the impacts. As traditional materials they considered PET and PLA as innovative packaging. A cradle-to-grave approach was followed, with the supply chain framework reported in Figure 8.

**Figure 9:**
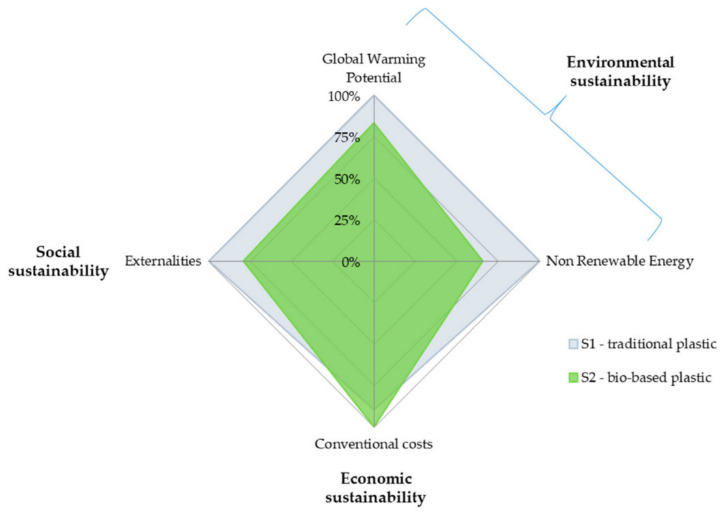
Sustainable performance results according to the triple bottom line (TBL) approach. Reprinted from [53].

As a functional unit a standard sale of 125 g flow pack was chosen. The results, reported on Figure 9 showed that bio-based plastics have lower environmental and social impacts than the conventional one, whereas the latter is the best choice according to a classic economic approach. The authors underlined as a success the use of bio-based material, instead of the traditional plastics (such as PE and PET), is linked to the ability to operate in a systemic way along the whole supply chain. 

In the same year, Giovenzana et al. [54] reported the results of a case study on the environmental impact analysis made on a new packaging solution used to oil quality preservation. This new packaging solution, a three layers packaging made from two bio-based polymers such as PLA treated with metallization and Bio-PE was compared with the traditional packages such as PE, PET and aluminum. An LCA cradle-to-grave approach was used, choosing as functional unit a single-use packaging olive oil content equal to 10 mL. With respect to traditional packaging, the innovative one obtained better performance in the human health impact categories but the fermentation process of sugar cane, used to obtain the PLA raw material, showed higher impacts in the ecosystem quality impact categories related to water resource depletion, freshwater eco-toxicity and land use. The improvement of environmental sustainability using an innovative packaging solution was not confirmed underline as the use of the bio-based product does not always represent the better way in terms of environmental sustainability, especially when recycling becomes an important aspect to be considered.

Finally, in 2020 Ferrara et al. [55] performed an LCA case study comparing the environmental performance of Italian wine packed in traditional single-use glass bottle and wine packed using four different packaging alternatives such as aseptic carton, bag-in-box, refillable glass bottle and multilayer PET bottle. All packages were chosen by the authors because they considered it suitable to contain wine while protecting it from light and oxygen during the storage time (considered within one year) and preserving its quality level. However, for a considerable length of time before opening (over one year), they reported clearly that the aseptic carton, bag-in-box and multilayer PET bottle cannot substitute glass bottles for high-quality aged wine [56]. All primary data were obtained by the packaging companies for weight, size and composition, for all packaging components (primary, secondary and tertiary packages), comprising also transport, distribution and final disposal scenarios. Secondary data were obtained from literature and technical documents. ReCiPe 2016 H was used as evaluation method, with both midpoint and endpoint approaches. From the results it was observed that the most environmental alternative was the bag- in-box packaging, slightly better than the aseptic carton, due to the composition of the containers, lower packaging weight and greater palletizing efficiency. Compared with single-use glass bottles, the impact of the bag-in-box was 60% to 90% lower. Considering as alternative scenarios the weight of containers, the wine distribution distance and the packaging disposal scenario, it was observed that decreasing the distribution distance, the environmental performances of refillable glass bottles could be comparable to those of the other two alternatives, pointing out as glass bottle reuse in Italy is a convenient alternative only when the distribution is performed at distances less than 100 km (local market). This study provided also useful indications for industry and government stakeholders for new strategies in order to enhance the sustainability of the wine life cycle, as well as countering the prejudice that glass packaging would be more sustainable than plastic or multilayer packaging.

### 2.2. Agriculture

Degradability and renewability have become one of the guiding principles for sustainable production and consumption of plastic commodities [2]. These two aspects have been documented to be largely satisfied by biopolymers: the latter have, indeed, been expanding mainly in response to the growing concerns about the sustainability of conventional plastics and to the environmental impacts caused by the uncontrolled disposal of plastic wastes [3]. Over the years, such polymers have been developed and improved in a way to become valid substitutes to the conventional ones, and so to find applications in a wide range of sectors.

In this regard, Shen et al. [57] identified the following biopolymers as utilizable in agriculture: starches; PLA; PHA; poly(butylene succinate) (PBS); PE and PVC produced from ethanol. Riggi et al. [2] reviewed their applications in crop production, and came to the conclusion that, by replacing their petroleum-based counterparts, these polymers may contribute to the greening of plasticulture. Furthermore, in their review study, Riggi et al. detected several research advances and patents, mainly based upon application of methodologies to expand the usage of biopolymers. This is the case of biodegradable spray mulching coatings from hydrolyzed proteins that resulted as enabling several benefits like: reducing mulch application costs; adding plant adjuvants in the sprayed mixture; and making spray mulching more versatile and utilizable where the conventional, LDPE-film based one is not [2,58].

Innovation in the mulch application field was researched also by Gorgitano and Pirilli [59], who explored the feasibility and environmental impact of using biopolymers as alternative film materials to the fossil counterparts. In particular, the authors’ attention was focused upon comparing LDPE to a Mater-bi (MBi)-based biodegradable polymer as mulch film in the growing of different greenhouse crops, namely lettuce, cantaloupe melon, and kohlrabi. For the study development, the authors carried out an LCA considering a set of impact categories to be consistent with the system investigated and best describe its environmental profile, including the potentials of global warming, acidification, and eutrophication, as extrapolated from the CML1 baseline. The Functional Unit chosen by the authors was 1 ha land where cultivation was carried out under greenhouse conditions using traditional and innovative mulching film, alternatively. The system boundaries were of the gate-to-gate type, and so encompassed all material and energy inputs, as well as all the activities carried out, from production to harvest. The study allowed the authors to understand that, on average, for all crops and impact categories investigated by the authors, MBi-based films perform better than the LDPE ones, but results are significantly affected by the mulch film dimensions and features, as well as by the given cultivation practices.

In another study, Russo and Scarascia Mugnozza [60] carried out a LCA of table tomato production, comparing traditional with hydroponic farming. In both cases, cultivation took place in greenhouse systems characterized by equal size (10 × 100 m^2^) but with different materials and structures. The FU was represented by 1 kg table tomatoes with the system boundaries going from production to harvesting and later distribution, including greenhouse maintenance and component replacement. With regard to the greenhouse system, for all impact categories considered according to CML 2 2000, a pitched-roof woody structure with an LDPE film cover was far more environmentally sustainable than a pitched-roof structure in zinc-coated steel with a glass cover and being more sustainable than a vaulted roof structure in a zinc-coated structure with LDPE as covering film. Therefore, it can be asserted that the combined utilization of wood and plastics enable more sustainable greenhouse systems but this solution was not feasible for a series of surrounding conditions and the steel-plastic mix system may be considered as the environmentally viable trade-off: as a matter fact, it was chosen by the authors to compare the conventional vs. the hydroponic cultivation. Related results from this part of the study have not been discussed here because they were considered by the authors as falling outside the scope of this review paper.

The studies reviewed found ways for the greening of plasticulture also through incremental innovation solutions, but there are still challenges to face, as related to background conditions, materials utilized and production processes, and sustainability assessment methodologies. Agreeing with Riggi et al. [2], those challenges can be solved through a multidisciplinary approach that combines the efforts of scientists and technicians with expertise in subjects like chemistry, engineering, agronomy and biology.

### 2.3. Automotive

**Figure 10:**
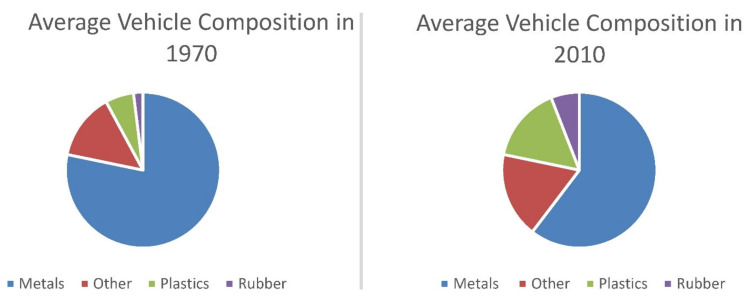
Change in vehicle composition from 1970 to 2010. Reprinted from [62].

The automotive industry has an important multiplier effect on the economy, because it is important not only for downstream industries but in particular for upstream industries such as chemical ones. Despite the fact that 80% of the growth in the sector is expected to occur outside the European Union (EU), it remains crucial for the economy of the old continent. 13.8 million Europeans (2.6 million directly) are involved by the car industry (6.1% of total European employment). But overall, the EU is at the forefront of investment in research and development (R&D) [61]. Thus the sector, in order to maintain and/or strengthen this leadership, in the last two decades has been searching for a sustainable approach in the design, manufacture and recycle/disposal of the materials employed in the automotive industry. The use of polymers and composite materials in the automotive industry has been increasing in recent years (Figure 10) both because legislative and consumer demands for lighter weight and fuel-efficient vehicles and because the design and complex parts of cars are more easily mouldable [62].

Based upon the subject literature, the sustainable development of supply processes consist of four levels, starting from the normative up to the monitoring of those processes, with their early detection and implementation in between [63]. Generally speaking, it is obvious that prevention is the key point to be taken into account for achieving a more sustainable production procedure, thus we cannot leave aside an analysis tool like LCA. 

Passarini et al. considered the increase in light material content such as polymers in new vehicle production, and hoping for an improvement of polymer separation according to an ecodesign-oriented strategy, applied the LCA to the end-of-life treatments [64]. The research group at Bologna “Alma Mater Studiorum” University, by considering the residual waste after shredding end-of-life vehicles (ELVs) aimed at proposing the trend of the environmental effects of treating an automotive shredder residue (ASR). They analyzed, according to the Ecoindicator 99 method (Pré Consultant, 2001) and using the SimaPro 7.1 software, integrated with Ecoinvent 2.0 database (Ecoinvent, 2008), the potential benefits from the recycling of polyolefins from ELVs, for the Italian situation. In fact, polymers’ growing share in the composition of new cars obliged to reconsider the EoL stage, trying to find effective recycling methods for polymers, which initially were sent to landfill due to the difficulty in being sorted and recycled with respect to steel and light metals. In particular, Passarini et al. evaluated how the different treatments (gasification, incineration, landfill, mechanical recycling) worked as a function of the time-dependent waste composition, showing the importance of LCA application as a tool for identifying a suitable waste management hierarchy, in accordance with the Directive 2008/98/EC. They concluded that recycling plants, modeling mechanical and chemical recycling options, achieved the lowest impacts due to the combination of material and energy recovery, with a consequent decrease in the residual amount of waste disposed of in landfill. Since the presence of recyclable materials and the net calorific value are fundamental parameters for the mass and energy balance during the Life Cycle Inventory (LCI) phase, they highlighted the need to consider their long-term evolving composition (ASR mix composition) in order to choose the most environmentally suitable technologies, especially when an investment in new technologies and plants, planned to be in operation for many years, is made.

**Figure 11:**
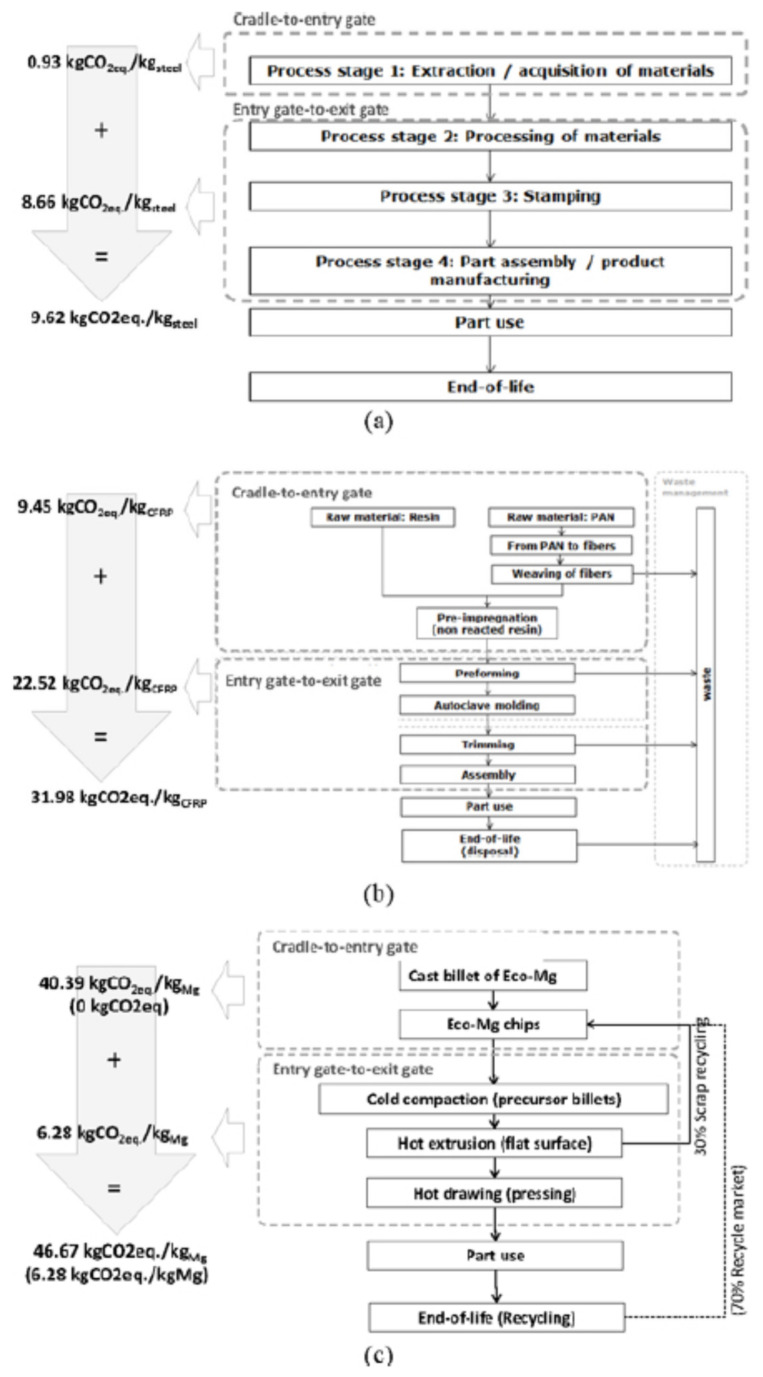
Process cycles and GWP results by Carbon Calculation over the Life Cycle (CCalC) software calculation: (**a**) LCA of conventional steel floor pan; (**b**) LCA of CFRP floor pan; (**c**) LCA for novel process route of extrusion of in-situ recycled Ecochips mixed with: (i) 70% of fresh material provided (cast billet and ecochips pathway); (ii) (in parenthesis) 70% of raw material entirely provided by recycling market. Reprinted from [65], © 2020 with permission from Springer Nature.

D’Errico and Ranza applied LCA to determine the environmental impact of an Eco-floor panel for cars in comparison with two other conventional manufacturing process cycles [65]. Namely, they compared a stamped steel floor panel, a polyacrylonitrile (PAN) carbon fiber blended with epoxy resin panel and a panel addressed by recycled eco-magnesium. They assumed a double scenario and reported the 3 process cycles and relative GWP per kg of material produced (Figure 11):

(i) 30% in situ recycled mixed with 70% of fresh material;
(ii) 30% in situ recycled mixed with 70% of recycled chips.

The purpose of the Milanese researchers was the evaluation of lightweight solutions in a cradle-to-exit gate stage modality, thus including the so called dirty-phase that is different for each considered material. The result of this approach was that, considering a car a lifespan of 200,000 km, carbon fiber reinforced polymer (CRFP) panels showed the best performance. On the other hand, novel Mg-based panels have not any technology limit in using 100% of recycled feedstock materials, thus allowing to align final GW values of low-impacting Eco-Mg produced by chips to those low values typical of a CRFP panel.

Ingarao et al. evaluated the environmental impact of some materials in means of transportation, by considering all their life cycle stages [66]. Their contribution aiming at improving the reliability of modeling the material (including recycling) and the manufacturing steps of aluminum-based components in comparison with those of glass-fiber-reinforced polymers (GFRP), considering light-weight benefits without neglecting the material production impact. Taking advantage of an industrial case study, coupling experimental measurements with industrial data and literature analysis, they carried out a life-cycle analysis of window panels to be assembled on the Italian high-speed trains. In particular, they focused on an innovative recycling strategy and manufacturing inventory issues. Concerning the functional unit, the life cycle of a single train window panel was analyzed. Each train, covering an annual distance of 500,000 km and an expected lifespan of 25 years, was considered for 112 panels, setting the FU as one of this panel. The system boundaries were settled from cradle-to-grave adopting the recycling as EoL strategy. 

**Figure 12:**
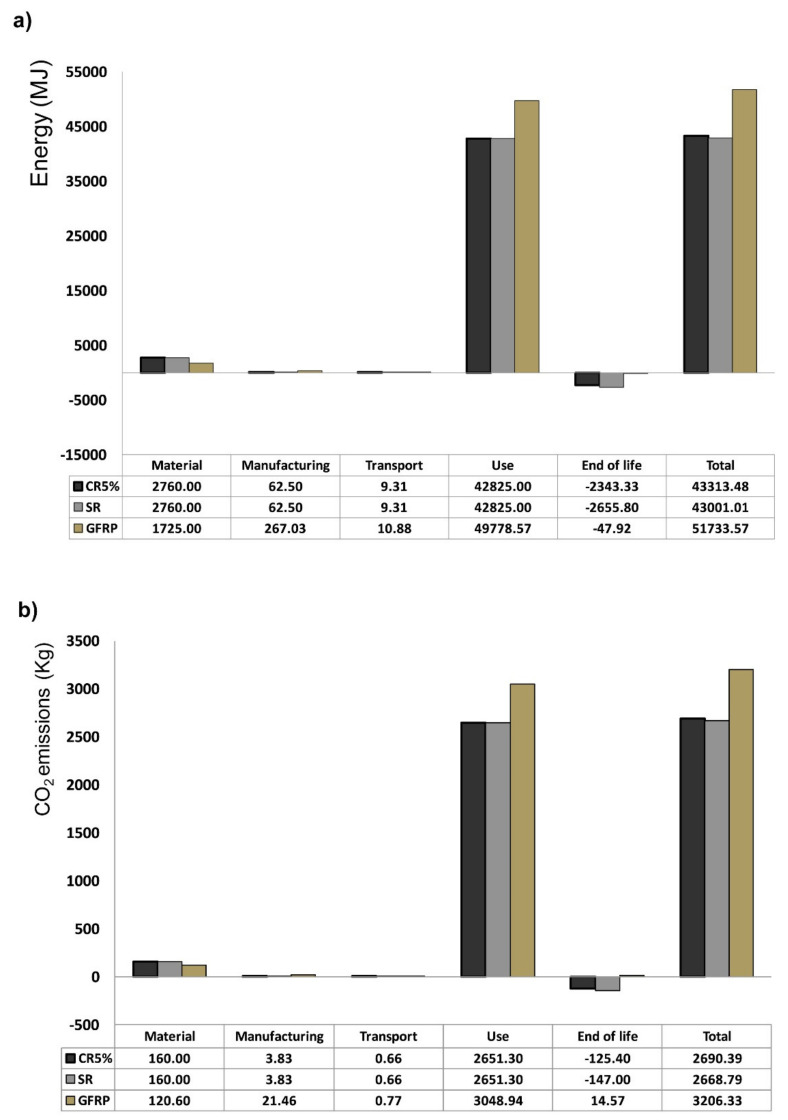
Comparative life cycle inventory results: (**a**) primary energy, (**b**) CO_2_ emissions. Reprinted from [66], © 2020 with permission from Elsevier.

The impact of material production, product manufacturing, transportation, product use and recycling were evaluated, analyzing primary energy and CO_2_ emissions flows throughout the panel life cycle. As shown in Figure 12, GFRP performed worst, both for CO_2_ emissions and energy demand, with the use that has proved, by far, the most impactful of the various phases. 

As regards the other phases, GRFP performed better during the material production (1725 vs. 2760 MJ and 120.6 vs. 160 kg of CO_2_), but subtracting the credit arising for recycling, the aluminum resulted in being by far the best solution for both the considered metrics. Finally, they concluded that for high-speed trains and in general for long service life transportation means light-weighting is definitely the strategy to follow, considering also that the long traveling distance allows for the possible worse environmental performances in material or manufacturing steps to be easily compensated.

**Figure 13:**
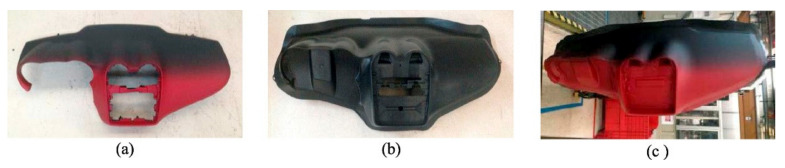
Automotive dashboard panel: finalized component (**a**); bottom layer (**b**); upper mantle (**c**). Reprinted from [68], © 2020 with permission from Elsevier.

Also Zanchi et al. evaluated, according to the LCA and LCC [67] methods, a light weighting strategies aiming at reducing impact during the use phase of a vehicle. They compared two different composite-based solutions for a dashboard panel manufactured by Magneti Marelli^®^, the first one reinforced with talc whilst the second based on the use of hollow glass microspheres (HGM), considering the environmental assessment and the economic sustainability [68]. The economic and environmental analyses were based on a detailed data collection (comprising primary data), including and comparing, in the first case, the production cost with the cost saving during the use phase, and, in the second case, different indicators aiming at highlighting the trade-off among manufacturing and use and possible EoL scenarios. They studied three different PP layers with two solutions differing in the bottom one (Figure 13), the first reinforced with 25% talc and the second one reinforced with 23% HGM. 

They settled as FU a dashboard panel of an Italian car (Alfa Romeo Mito 955 diesel engine) having a lifespan of 150,000 km/10 years. The system boundaries included materials production, component manufacturing, transport of materials and of the finalized dashboard to plant for its assembly to the vehicle, use phase and EoL treatments. Results showed that HGM-reinforced composite was likely better from an environmental point of view for those impact categories where the use phase is more involved. The increase of material processing impact did not compromise benefits in terms of GWP and primary energy demand due to weight reduction, nevertheless it affected resource depletion and ecotoxicity indicators negatively, but overall, the EoL phase was not affected significantly. They thus concluded that, despite a higher material cost, the use of HGM reinforced composites was found economically preferable as demonstrated also by the breakeven point and it was suitable for mass customization.

Zanchi et al. continued their LCA investigation extending the previous methodological approach in the design of other Magneti Marelli^®^ components by using GaBi software [69]. The various automotive components were sorted by three vehicle systems: drivetrain (namely: air intake manifold, throttle body, muffler, fuel tank); interior (dashboard, pedal box support, brake pedal); Suspensions and chassis (Crossmember, Suspension arm). Each component was analyzed in terms of elementary flows and products/waste flows having as goal and scope the determination of the environmental performances along the entire life cycle of innovative lightweight solutions when compared with the current heavier designs. They pointed out the difficult of benefits prediction when lightweight polymeric materials were used in the place of heavy metal ones, again for the trade-off among manufacturing and use. Thus, they concluded their efforts in this field with a series of take-home messages:

- A closer synergy between the various experts involved in the design phase is necessary to select materials that actually affect the reduction of impacts, thus enhancing increasingly accurate data collection and environmental modelling.
- The introduction of different impact categories, then GWP could make the results interpretation more difficult. They recommended ensuring an updating process of internal LCA expertise concerning continuous scientific progress in the LCA fields regarding methods to calculate and interpret the impacts.
- The assumptions on the final treatment of ASR (landfill or incineration) and recycling rate values become significant for a complete substitution of heavy metals with polymer-based materials.
- They recommended including secondary effects in the LCA comparison to obtain more precise results concerning benefits from lightweight design solutions.

**Figure 14:**
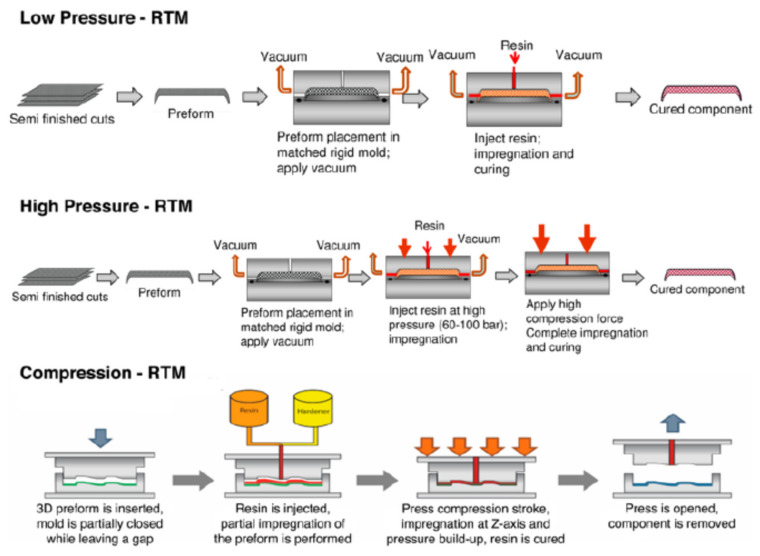
Resin transfer molding (RTM) variants analyzed. Reprinted from [70], © 2020 with permission from Elsevier.

The production of light and high-performance components for the automotive was also the object of the study of Vita and his colleagues [70]. The researchers of the Universities of Ancona and Viterbo, by the means of LCA, compared the environmental impacts of three alternative resin transfer molding (RTM) manufacturing processes for the production of CFRP car hoods: namely Low Pressure-RTM (LP-RTM), High Pressure-RTM (HP-RTM) and Compression-RTM (C-RTM) (Figure 14).

**Figure 15:**
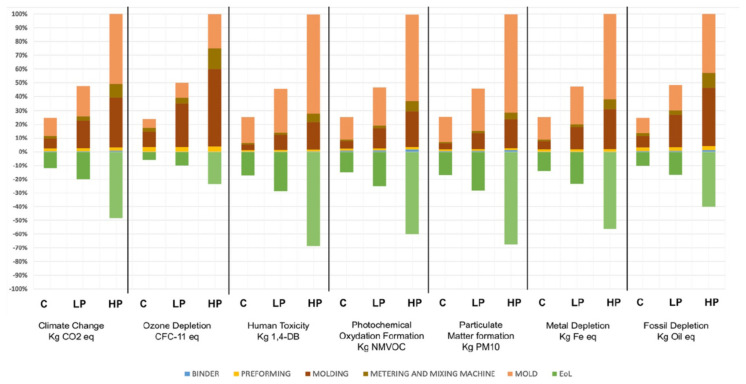
Comparison of the RTM variants in terms of ReCiPe mid-points. Reprinted from [70], © 2020 with permission from Elsevier.

A car hood, mounted in an Italian luxury car, was taken as reference part and the functional unit was defined as the production of 1000 CFRP car hoods through the three processes under investigation. The system boundaries included the material extraction, manufacturing and EoL phases for those flows directly involved in processes. The LCA analysis was carried out using SimaPro 8.05.13 as software and the database EcoInvent 3.1 as the supporting inventory database. They observed that the mold size directly influenced the energy consumption of the heating and cooling systems, thus it was evident that moving towards low-pressure methods, such as C-RTM or LP-RTM, led to a drastic reduction of impacts caused by the manufacturing of CFRP components (Figure 15). Anyhow, a reduction of the electricity in these systems could be desirable, for example by using more efficient heating methods and materials.

**Figure 16:**
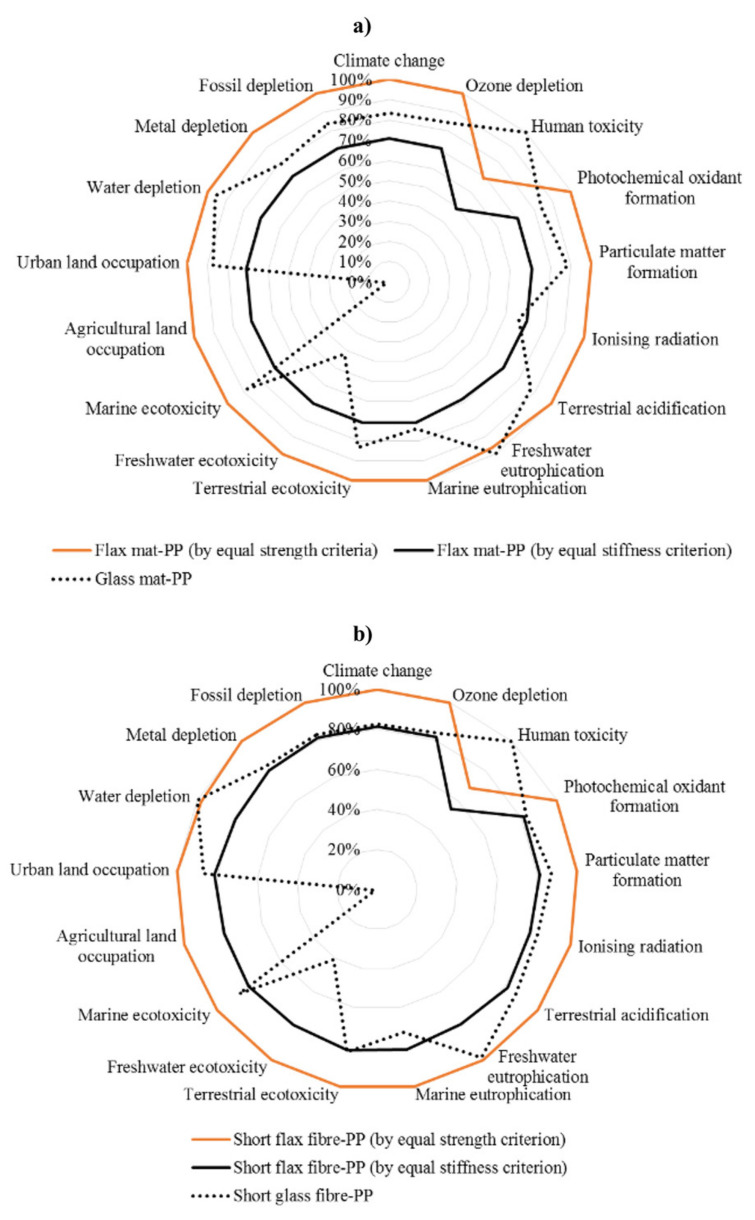
Life cycle impact comparison between flax fiber-reinforced polymers (FRPs) and glass fiber-reinforced polymers (GFRPs) with equal stiffness and equal strength design criteria. (**a**) Flax mat-PP. (**b**) Short flax fiber-PP. Reprinted from [71], © 2020 with permission from Wiley.

Ingarao and some foreign colleagues, combining the generalized rule-of-mixture (ROM) model and the Ashby material selection one, compared flax-fiber reinforced polymers (FRPs) and GFRPs as lightweight components for automotives [71]. The purpose of this research group was to assess, by the means of LCA, the cradle-to-grave environmental impact of flax FRPs with different design specifications (fiber format, volume fraction, manufacturing technique, and load-bearing capacity) and comparing to those of the conventional GFRPs. The functional unit was designed as 200,000 km driving distance during the lifespan. Two types of flax FRPs, injection molded short flax fiber reinforced polypropylene (short flax-PP) and compound/compression molded flax mat polypropylene (flax mat-PP) film, were investigated and compared with conventional glass mat-PP and short glass fiber-PP. They evaluated multiple impact categories and incineration with energy recovery was chosen as the EoL scenario for both the composites. The flax mat-PP showed, under the equal stiffness criterion, a lower environmental impact than a glass fiber-mat in most of the impact categories (Figure 16), due to its lightweight structure, however, flax mat-PP composites suffered from environmental issues common to agroproducts. By contrast, considering the dominant impact categories, the total life cycle impact of the short flax fiber-PP was very similar to that of the short glass fiber-PP. Finally, since the fiber volume fraction can be considered the critical parameter they evaluated its influence on the impacts, showing the best volume fractions (28–32% v/v) for the flax mat-PP composite differently than flax fiber-PP which exhibited steady decreased life-cycle CO_2_ emissions. 

### 2.4. Concrete-Polymeric Materials

Concrete-polymer materials are extensively used due to their unique properties leading to an extended service life, in particular in the structural applications. The major causes of deterioration of reinforced concrete structures, such as the corrosion of the steel reinforcement, industrial chemicals, deicing salts, and moisture, can be overcome by using FRP bars as an alternative type of reinforcement [72,73]. 

Redaelli et al. carried out a LCA sustainability analysis on a prototype culvert made of different type of concrete and reinforcement (carbon steel, austenitic and duplex stainless steels, and glass fiber–reinforced polymer) providing a comparison among the different typologies [74]. The analysis was firstly carried out from cradle to the gate (i.e., up to culvert installation) by assuming a 5 m culvert segment as FU, for the LCI and GFRP were considered primary data by the industry partners, whilst for the remaining materials and processes were used secondary data from the Ecoinvent database. Among the different concrete formulations, that containing carbon steel (CS) resulted the cheapest to the gate. They successively extended their study to the operational and EoL stages of the infrastructure, by performing cradle-to-the-grave analysis over a period of 100 years. In these new boundaries, the formulation containing CS resulted the worst one, both in terms of environmental impact and cost, because of the additional required maintenance. The formulation used glass fiber–reinforced polymer reinforcements proved to be the cheapest solution and, as regards the environmental impacts, the most sustainable solution, followed by the stainless steel. The LCA results, among the different formulations, did not change when the study period was decreased to 50 years. 

A LCA and also a LCC analysis on four non-corrosive reinforcement materials for concrete structures were carried out by Dotelli et al. taking into account stainless-steel (SS); GFRP rebars, CFRP strands and epoxy-coated steel (ECS) reinforcing bars [75,76]. SIMAPRO (version 8.5.2.0, 2018) software and Ecoinvent database, as source of secondary data, were used firstly in a cradle-to-gate (raw materials, transportation, and construction) investigation and then in a cradle-to-grave (raw materials, transportation, construction, maintenance, and EOL) one. The selected impact categories were GWP 100, ozone-depletion potential (ODP), acidification potential (AP), photochemical oxidant creation potential (POCP) and eutrophication potential (EP). CFRPs reinforced formulations outperformed in four out of five categories, resulting in not being environmentally competitive only for the ozone depletion. Concrete reinforced with SS well performing in three out of five categories, resulted in being the first alternative to the fiber-reinforced polymer. Anyway, due to the large contribution in terms of global warming of the SS’s reinforced concrete (plus 35,508 kg CO_2_ eq.) the former formulation remained the largely preferable choice. Their results indicated the ECS reinforcing bars as the least environmental- friendly solution, since there was not a single category for which it was an ideal alternative. Also as regards the demolition and landfill activities at EoL, the results of Dotelli and his colleagues indicated the FRP formulations as the best option, because did not require major operations (such as cathodic protections or reinforcement replacement), and, thus, did not require traffic disruptions or traffic diversions. 

The comparison between the use of FRP and steel in reinforcing the masonry buildings was also the subject of the study carried out by Russo et al. at the University of Venice [77]. They evaluated the LCA of the two options when using as retrofit work to increase the overall strength of masonry structures against seismic actions. They observed that, in terms of sustainability, costs and environmental impact, the weight of steel and the need for maintenance can become a critical problem, thus proposing as a sustainable solution the use of FRP profiles instead of the steel ones. 

**Figure 17:**
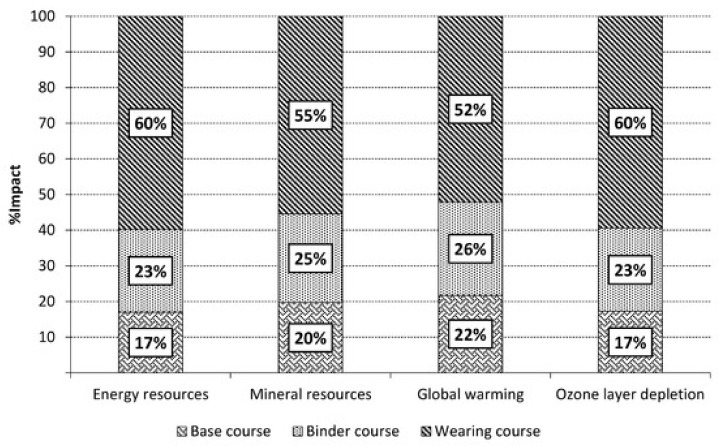
Contribution of the pavement layers to selected impact categories, scenario S. Reprinted from [78].

Puccini et al. approaching to a sustainable design and management of transport infrastructures applied the LCA tool to study different solutions of low noise pavements. Their idea was based on replacing, in the bituminous mixtures, the classic virgin non-renewable materials with secondary materials to lower landfill pressure, maintaining adequate functional properties [78]. Thus, the classic hot mix asphalt (HMA) was compared with crumb rubber (CR) from EoL tires and reclaimed asphalt pavement (RAP). They carried out a cradle to grave study, from production to EoL stages, including laying and maintenance in an Italian urban road located in the municipality of Massarosa in the province of Lucca (Tuscany). They settled on 400 m and 464.5 m of pavement of the road as FU and the data collected were analyzed by SimaPro 8.3.0 software. The researchers of the University of Pisa demonstrated how the use of recycled materials leads to a 50% reduction of the environmental burdens, confirming the high impacts associated with the use of the classic HMA mixtures (Figure 17). 

Some of the observed reduction (material depletion and energy consumption) was attributed to the different level of maintenance requested. As regards the reduction in raw materials and hydrocarbons emission was attributed to the use of reclaimed materials. Thus, the combined use of recycled materials and warm technologies was indicated as a methodological approach to improve the environmental sustainability of low noise pavements. 

### 2.5. Geopolymers

In the previous paragraph we got to see how, considering concrete the most commonly used construction material worldwide, many efforts were carried out in recent years to improve their functional properties trying to increase, in the meantime, its environmental performance. In fact it is well known that this huge amount leads to a relevant environmental impact, concrete occupy large volumes in landfills, and its manufacturing is highly energy intensive. Cement industry can be considered among the largest CO_2_ emission sources [79]. For the above cited reason both sector industry and academy devoted their studies to eco-sustainable materials, such as geopolymers, as components of a new class of low-energy materials maintaining the specific properties of cements [80]. As we reported for concrete and also for geopolymers LCA represents the most common approach to assess potential environmental impacts, showing that the inclusion of industrial waste provides the enhancement of its environmental performance and the reduction of costs. In particular, the recycling of waste, by using in their formulation slags, calcined clays and coal fly ashes [81,82,83,84], leads to a reduction in energy and natural resource consumption.

A LCA investigation, concerning the environmental burdens related to the use of wastes from recycling construction and demolition treatment plant as raw materials in the geopolymer’s production, was carried out by Petrillo et al. regarding the production of concrete blocks in plants located in the South of Italy [85]. Their study referred to different plants located in the Campania Region (South of Italy), starting from raw materials acquisition and processing up to the production and packing of concrete paving blocks, thus reporting results in a cradle-to-gate approach. The ordinary Portland cement (OPC) block were obtained by mixing cement with recycled clay soil as solid precursors and blast furnace slag, designing as functional unit 1 m^2^ of paver blocks. The various life-cycle stages of brick products were modelled and assessed with SimaPro VC LCA software, by using Eco-indicator 99 and CML 2000 baseline. 

**Figure 18:**
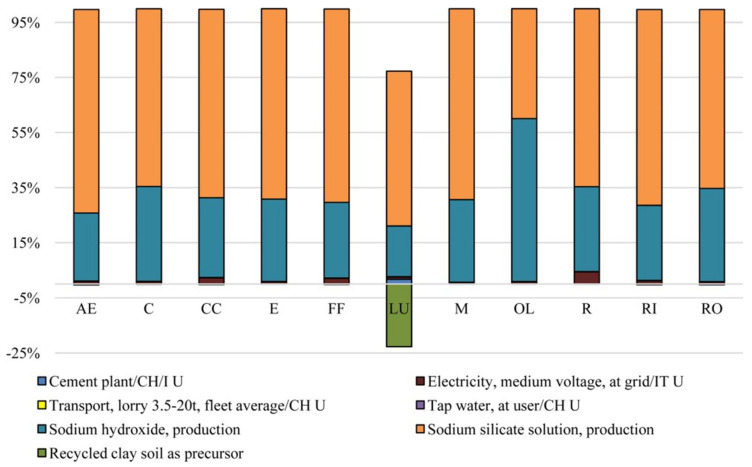
ECO 99 characterization values of 1212 kg of geopolymeric binder (AE: acidification/eutrophication; C: carcinogenics; CC: climatic change; E: ecotoxicity; FF: fossil fuels; LU: land use; M: minerals; OL: ozone layer; R: radiation; RI: respiratory inorganics; RO: respiratory organics). Reprinted from [85], © 2020 with permission from Wiley.

In both the methods used, they indicated the binder and concrete production processes as the hot spot for energy consumption and thus the main responsible for CO_2_ emissions (Figure 18).

**Figure 19:**
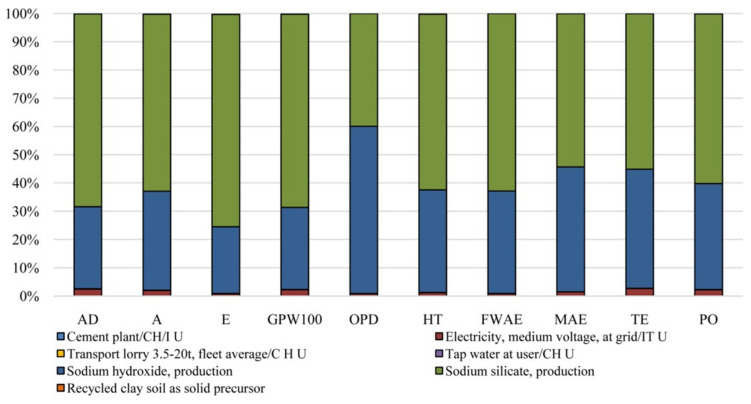
CML characterization values of 1212 kg of geopolymeric binder (AD: abiotic depletion; A: acidification; E: eutrophication; FWAE: fresh water aquatic ecotox; GWP: global warming potential; HT: human toxicity; MAE: marine aquatic ecotoxicity; ODP: ozone layer depletion; PO: photochemical oxidation; TE: terrestrial ecotoxicity). Reprinted from [85], © 2020 with permission from Wiley.

Other relevant impact categories, involving the use and production of alkali activators, were the ozone layer depletion and eutrophication ones for the CML method and respiratory inorganics and ozone layer depletion for Eco-indicator 99 (Figure 19). They concluded that the use of recycled clay soil or waste for making paving blocks would address sustainable issues such as the resource conservation and conversion of wastes and byproducts to useful and valuable products, although an LCC analysis could be necessary.

**Figure 20:**
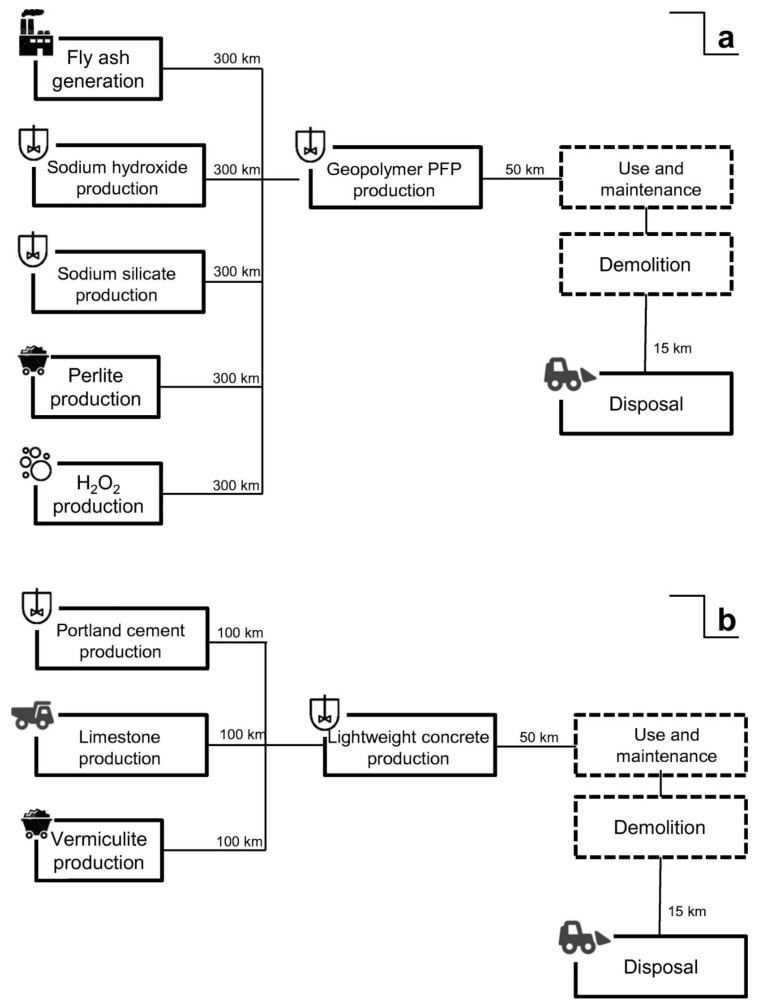
Main unit processes considered for (**a**) geopolymer based passive fire protection (PFP) mix and (**b**) lightweight concrete PFP mix in case B. Dashed box: processes outside system boundaries. Reprinted from [86], © 2020 with permission from Springer Nature.

Tugnoli et al. carried out a LCA analysis aiming at evaluating the replacement of the conventional cementitious binders in the passive fire protection (PFP) systems with geopolymer-based matrices [86], because of their high thermal stability and low thermal conductivity [83,87]. They compared the environmental and economic performance of a PFP geopolymer with a commercial cement, having similar fireproofing properties, that was used as reference. The research group of the University of Bologna divided the LCA study into two cases, A and B respectively. In the former they investigated a geopolymer-based OPC replacement block, by designing a geopolymer concrete block with 1 m^3^ as FU, and comparing it with data obtained in previous studies. In the second case, they investigated a geopolymer-based mixture (waste fly ash from the flue gas treatment of a coal-fired power plant as a precursor and Na-based alkali activators) for PFP application with the purpose to define its environmental footprint. To this aim and to make possible the comparison, they designed as FU an insulating PFP layer of 66.5 m^2^. The analyses were carried out from cradle-to-grave with secondary data coming from the Ecoinvent database 2.0 and the thinkstep free GaBi LCI datasets. The main unit processes for both cases are reported in Figure 20, where the considered transportation distances are reported as well. 

**Figure 21:**
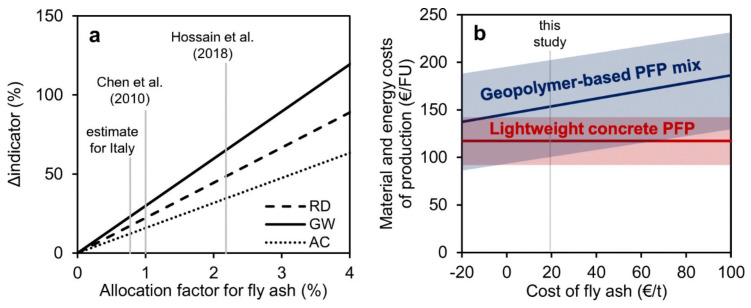
(**a**) Variation of the main environmental indicators for the geopolymer-based PFP mixture as a function of allocation of the coal life cycle impacts to the fly ash. (**b**) Estimated material and energy costs for the geopolymer-based mixture and for the lightweight concrete alternative as a function of the cost of fly ash. Reprinted from [87], © 2020 with permission from Springer Nature.

By making a balance among the avoiding of CO_2_ emissions from the clinker process in cement manufacturing and the alkali activation of the geopolymers (affecting relevant parameters like AP, EP) the proposed new geopoloymeric systems performed a life-cycle impact which was 27% lower than that of the reference in terms of GW. These results confirmed the overall sustainability and the economic viability (Figure 21) of geopolymers for PFP applications with the authors’ recommendation in employing sodium silicate produced via the hydrothermal route and in partial substitution of sodium silicate with waste-derived alkali activators.

Aiming at demonstrating the feasibility of the production of aerated systems based on geopolymer hybrid foams (GHF), derived from fly ash (GHF-FA), Roviello et al. applied LCA to evaluate the impacts of this process in comparison with GHF based on metakaolin (GHF-MK) and commercial aerated autoclaved concrete (AAC) systems [88]. In the authors’ intentions the data deriving from LCA must allowed to overcome the issues that, at that time, limited the geopolymers commercialization. To reach this goal they emphasized the inexpensiveness of the raw material and processes. 

**Figure 22:**
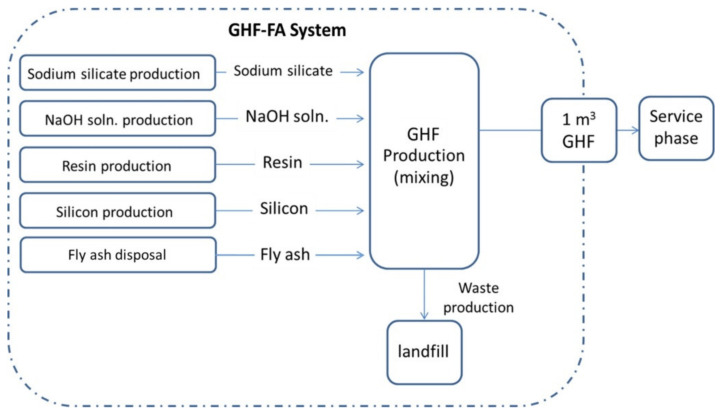
Description of geopolymer hybrid foams derived from fly ash (GHF-FA) life-cycle production. Reprinted from [88], © 2020 with permission from Elsevier.

In particular, they aimed at developing an eco-friendly process that, using waste raw materials, adopted the circular economy philosophy. They enclosed in their study raw materials, transport, manufacturing and disposal, thus considering a cradle-to-gate approach (Figure 22). LCA was carried out by the means of the SimaPro© software v.8, designing as FU 1 m^3^ of GHF and by using for primary data available datasets and literature data whilst, for secondary data, the Ecoinvent v.3 database, literature review and expert judgment. As expected, Roviello and her colleagues found that showed the best environmental performance paying, however, in terms of mechanical properties with respect to the systems. Thus, they demonstrated the feasibility of GHF-FA industrial production highlighting, however, the need for further studies to make comparable its usage performance to that of GHF-MK. 

### 2.6. Polymeric Materials in Fuel Cell

It is well known that automotive sector, from production to the road, contributes substantially to GHG emissions and air pollution [89,90]. In one of the previous paragraphs, we dealt with the production assessment, whilst in this one we face the problems related with the automobile’s use. The GHG emissions from road transport accounted for 18 and 28% in EU and USA, respectively [91,92] and in the last decade battery electric vehicles (BEVs) were proposed as environmental alternative to the fossil-based internal combustion engine vehicles (ICEVs) showing, however, disadvantages as regards short driving range and long refueling time. For this reason in the last years a technology, already developed in the 1960 s [93,94], like that of fuel cells (FC), has been reconsidered [95]. Various LCA analyses were carried out focusing mainly on the fuel cycle and accounting for roughly on the production and disposal of the cars [90]. 

**Figure 23:**
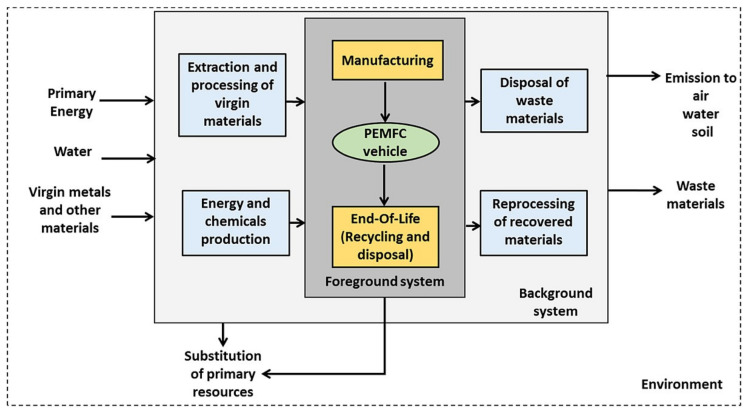
System boundary of the whole life cycle assessment of a polymer electrolyte membrane (PEM) fuel cell passenger vehicle. Reprinted from [96], © 2020 with permission from Elsevier.

Thus, in moving towards the understanding of the real environmental impacts of producing FC vehicles, in comparison to the classic or alternative green cars, and overcoming the limitations of existing studies, Lettieri et al. focused, by the means of LCA tools, on the manufacturing stages of a polymer electrolyte membrane (PEM) FC and compared the results obtained with those relating the production of BEV and ICEV [96]. They carried out a LCA analysis of FCV, ICEV and a BEV, respectively, considering their whole life cycle, including the manufacturing of the vehicles, their use phase and end-of-life (Figure 23), assuming a lifetime of 150,000 km for all three vehicles and a functional unit of 1 km driven by one car. 

**Figure 24:**
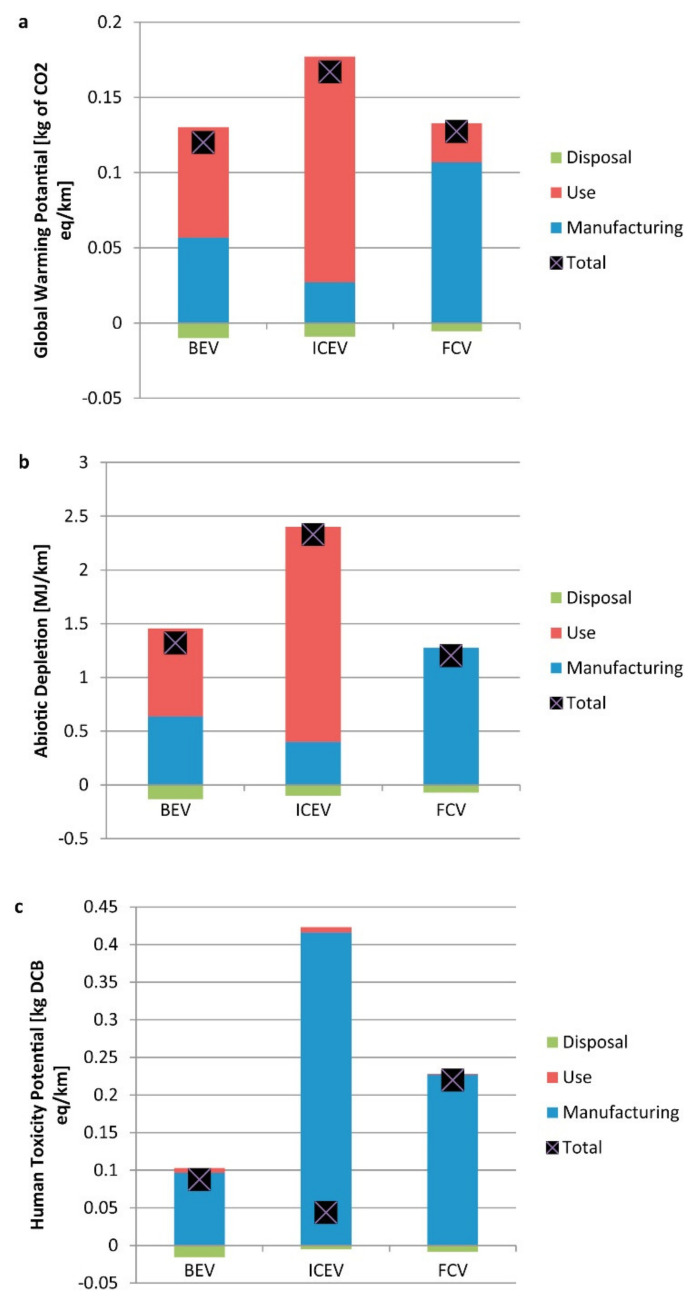
(**a**) Global warming potential, (**b**) abiotic depletion, (**c**) human toxicity potential of fuel cell vehicle (FCV), battery electric vehicle (BEV) and internal combustion engine vehicle (ICEV) for the whole life cycle. Reprinted from [96], © 2020 with permission from Elsevier.

The results obtained showed different environmental impacts of the three different vehicles as a function of the stages considered. FCV performed worse in the production process because of the hydrogen tank and the fuel cell stack, despite the scenario proposed suggested a 25% potential reduction in the climate change impact category. By contrast, analyzing the use stage FCV performed well in respect to ICEV, showing the highest GWP due to the fossil carbon emissions associated to the use of diesel (Figure 24). 

The GWP of the disposal stage was shown negligible for all three technologies, thus according to the authors FCV showed a significant improvement in the use stage but reducing the environmental impact associated with its manufacture still represents an important challenge that need to be addressed in future years.

Di Marcoberardino et al. after designed and tested a novel micro-combined heat and power (CHP) system based on membrane reactor and PEM fuel cell [97,98] proceeded with their study evaluating the environmental performance of the system, by using LCA, and the economic assessment to make it sustainable and cost-effective over its lifetime [99]. 

**Figure 25:**
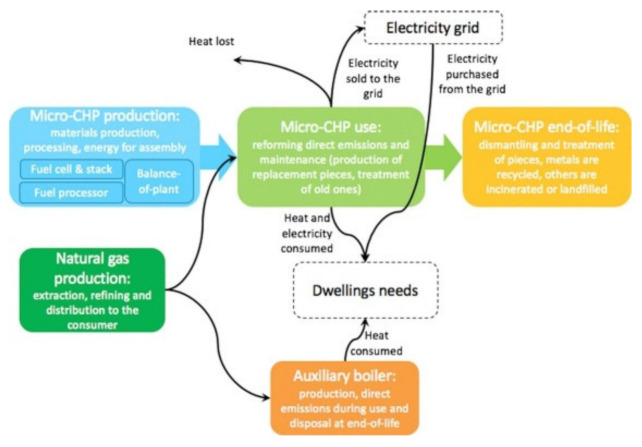
System boundaries considered for the LCA for the assessment of the steam reformer (SR) and autothermal reformer membrane reactor (ATR-MR) systems. Reprinted from [99], © 2020 with permission from Elsevier.

In particular, they focused their analysis on aspects such as process intensification and optimized thermal integration thus trying to reach a reduction of the material requirements, considering also aspects beside greenhouse gas emissions: water withdrawal, resources depletion, human health. They compared their innovative FC with a commercial PEM for various residential applications, supplied with conventional natural gas from electric grids in two different countries (Italy and Germany), thus making the assessment as generic as possible. Italy and Germany were chosen to compare different loads because of the different climate, and different natural gas and electricity costs. The evaluation was carried out, taking into account the full life cycle needed to provide heat and electricity (Figure 25), and assigning the functional unit as “provide one, two or 10 German or Italian dwellings with useful heat and electricity produced by a 5 kWel PEMFC micro-CHP over one year”. The LCA software used was IMPACT 2002+ v2.2 considering the following indicators: carbon footprint, resources, impacts on ecosystem quality, water withdrawal and impacts on human health. Among the results, whilst the impacts on the water withdrawal and human health can be positive or negative depending on the case, the innovative system undoubtedly reduced the carbon footprint impact, thus becoming competitive, from an economic point of view. 

### 2.7. Various Life-Cycle Assessment (LCA) Applications on Polymers

Given the specific applications we have seen in the above reported sections, in general in the last decade LCA has been applied to explore new design routes, processes and final treatment of polymer or polymer-based materials. The main goals that the authors addressed in the last paragraph was the reduction of the environmental impacts, proposing a different strategy, by the help of LCA, compared with the standard one. Bio-based polymeric materials in particular have attracted the attention of those researchers aiming at improving their environmental performance. 

**Figure 26:**
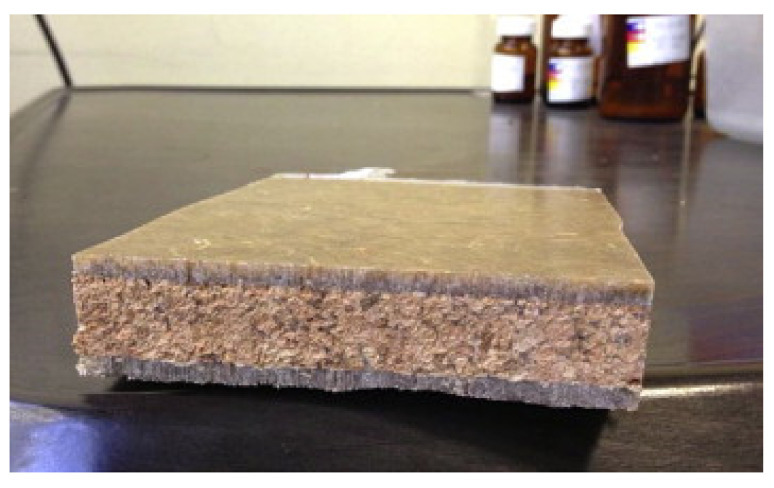
Eco-sandwich with core in granulated cork and skins in hemp/bio-resin. Reprinted from [101], © 2020 with permission from Elsevier.

La Rosa et al., by means of LCA, explored the possibility of improving the eco efficiency of glass fibre composite materials to be applied on a pipe system used to transport cooling sea water in a Sicilian petrochemical company [100]. In order to replace part of the conventional fibres with hemp mats a comparative LCA was performed on two different elbow-fittings made of glass fibre/thermoset composite and hybrid (glass fibre-hemp)/thermoset composite, respectively. The study was carried out from cradle to grave by considering as FU one elbow fitting used in the chemical plant pipeline, with an estimated life of 20 years and using data collected from the process as primary and the Ecoinvent v2.2 database for the secondary ones. They found an increase of the eco-efficiency for the hemp mat-reinforced composites, particularly during the production phase due to the ‘green’ origin of the hemp mat. The same authors, by using the Simapro 7.2 software, carried out a cradle-to-manufacture study in order to compare the production of an eco-sandwich panel containing cork, hemp and bio-based epoxy resin as natural materials with respect to a traditional sandwich made of epoxy resin and polyurethane/glass-fibers [101]. Aiming at evaluating the environmental impacts related to their production, they settled on an eco-sandwich panel sized (0.400 × 0.400 × 0.02 m) as FU (Figure 26).

LCA results showed a good improvement in terms of environmental impacts and energy use compared with the petroleum-based products, despite the major contributions to the impact (in both cases, eco-sandwich and traditional sandwich) being due to the use of epoxy resin (environmental impact up to >85%).

Lettieri et al., in the direction of a greening of the epoxy based materials, carried out a cradle to gate study comparing bacterial cellulose (BC)- and nanofibrillated cellulose (NFC)-reinforced epoxy composites with PLA and 30 wt.-% randomly oriented GFR/PP composites [102]. 

**Figure 27:**
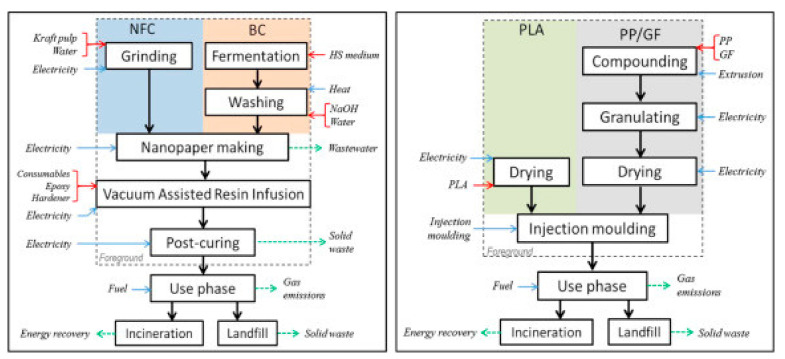
Schematic diagram showing the system boundaries of the model representing the life cycle of bacterial cellulose (BC-) and nanofibrillated cellulose (NFC)-reinforced polymer composites (**left**), and PLA and glass-fibre/polypropylene (GF/PP) composite (**right**). The red, blue and green arrows represent consumables or raw materials required, energy input and waste (materials and energy), respectively. Reprinted from [102], © 2020 with permission from Elsevier.

They assumed 1 kg of NFC-reinforced epoxy composite as FU and used version 6 of the GaBi software. The adopted model, developed by the Centre for Environmental Science in Leiden University [103], in order to minimize uncertainties provided midpoint indicators to model the effects of substances on the environment at an early stage (also known as the problem-oriented approach). Their research highlighted good mechanical properties for the proposed systems with respect to neat PLA, but in the meantime a higher GWP and abiotic depletion potential of fossil fuels of BC- and NFC-reinforced epoxy composites. They observed that by enlarging the boundaries to the grave (Figure 27) and increasing the nanocellulose content up to 60%, the GWP and abiotic depletion potential of the innovative composites could be lower than neat PLA, suggesting higher nanocellulose loading to reach the desired “greener credentials”.

A couple of years since their first studies, La Rosa et al., continuing LCA investigations on epoxies, proposed the idea of the recyclable composites production by engineering a commercial amine-based epoxy curing agent. After the manufacturing and the chemical characterization of this CF/epoxy composites, they evaluated the environmental impacts associated with the recyclability of the thermoset composite [104]. Thus aiming at highlighting the benefits of using Recyclamines^®^ in the production of structural fibre reinforced composites. The Ecoinvent database was used for the data and a CF/thermoset panel, manufactured through high-pressure (HP) RTM and chemically treated in order to recover clean CFs as well as an epoxy thermoplastic, was designed as FU. The first LCA results showed the environmental advantages in recovering clean CFs as well as a thermoplastic polymer that can be reused and re-processed. In a further study, they tested the efficacy of the recycling process, developed by Connora Technologies (Hayward, CA, USA), at the plant level, highlighting the great innovation introduced by Connora Technologies that induced the conversion of thermosets into thermoplastics [105]. The research group at the University of Catania obtained a series of LCA results, demonstrating a very low environmental impact associated with the process and the avoiding of furthermore impacts due to the recovery of the epoxy-composite constituents (fibres and matrix).

People working in manufacturing are always searching for new materials and process optimization to guarantee quality and safety standards. Gagliardi et al. focused their research on the evaluation of the impacts associated with the different joining processes (i.e., mechanical, thermal and chemical) involved in the assembling of materials, with particular regard to the hybrid ones [106]. They carried out cradle-to-gate and cradle-to-grave analyses but not considering the use phase and focusing mainly in the recycling aspects. In the first part of the study, the energy consumption and CO_2_ emissions of the investigated processes, showed the best environmental performance for the mechanical fastening performed by bolts. When they enlarged the boundaries, considering the recycling, the adhesive bonding and ultrasonic spot welding became environmentally friendly as well. 

**Figure 28:**
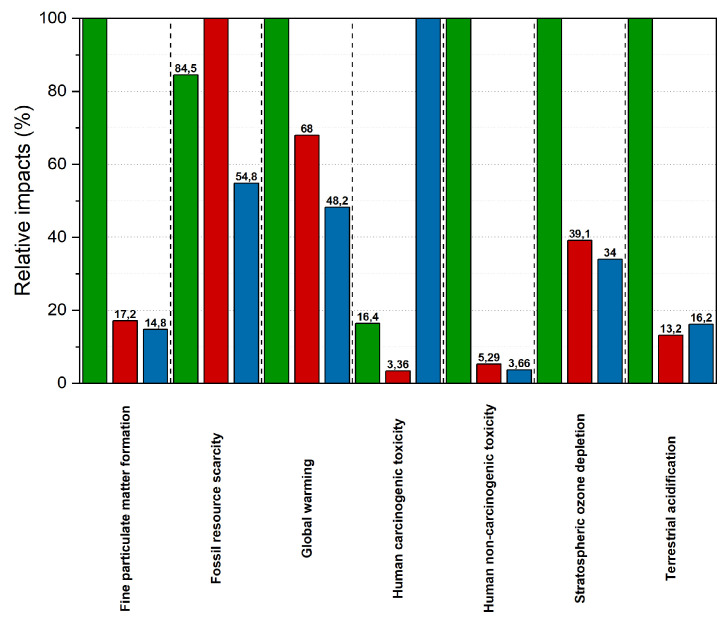
The histogram showing the relative impacts for the main and most reliable factors studied. Data were normalized to the maximum value for a rapid evaluation of the advantages and disadvantages of each material. Green bars refer to the impacts calculated for lead, red bars for the composite samples and blue bars are referred to steel impacts. Reprinted from [107].

The lowering of the environmental impacts and toxicity for human health in the design and production of materials for X-ray shielding applications was explored by Milanesio et al. [107], who proposed a new epoxy based composites filled with barium sulfate and bismuth oxide instead of the traditional screens made of lead and steel. The low-cost lightweight shielding composite materials was tested by LCA, from cradle to gate, by using openLCA 1.9 and the Ecoinvent 3.5 database. The proposed new epoxy-based panel performed better than both the traditional panels, in terms of GW and particulate formation with respect to the lead one and in terms of human health with respect to the steel one (Figure 28).

## 3. Future Outlooks

The fair amount of works analyzed in this review allowed the authors to understand not only that in Italy are LCA applications held in high regard, but, also, to draw considerations and look at the multitude of perspectives that LCA can offer to different sectors dealing with the design, production, marketing and final treatment of polymeric materials. As regards food packaging, the most investigated sector in Italy for number of manuscripts, application of LCA was double addressed. On the one hand, towards the aspects inherent the final treatment of the plastic materials to mitigate, or completely remove, the dispersion of the packaging in the environment, and on the other hand to replace the raw material to make it free from oil derivation. In the next few years, the authors expect a growing interest in the first of the two aspects, aiming at reducing the persistence of packaging in the environment. As far as they are concerned, the authors will continue working in this field of research by evaluating the relevant environmental impacts, and the consequential damages, that are associated with naturally derived polymers with high barrier properties for optimal food conservation levels. 

Furthermore, agriculture is a very sensible research area, especially in Mediterranean regions, like Sicily, where there is a massive consumption of plastic materials for different purposes, from mulching to greenhouse covers, through irrigation systems. Despite this, the authors found that very little has been done thus far at the environmental level compared with the other areas reviewed. This stimulates expansion of research on the subject to contribute to greening not only plastics’ life cycles but also the agricultural systems where those plastics’ are utilized. With regard to this point, this paper’s team of authors intend to co-work in the direction of developing and testing innovative plastic formulations that are compatible with the soil and/or enable improving its properties, in order to find the truly feasible ones. Experimental research will then be complemented with LCA to validate those formulations from an environmental perspective. 

Similarly, in the building sector, new concrete formulations can be designed using recycled plastics, without compromising the structural properties of the artefact. 

The review highlighted LCA to be an irreplaceable tool being able to couple economic, environmental and performance considerations thus leading to discovery of the most high-performing solution or, at least, the viable trade-off. 

Last but not least the automotive sector is historically a very important sector of the Italian economy. Here, LCA practitioners can indulge themselves, both for the innumerable polymeric components to be studied and for the possibility of extending or restricting the boundaries, consequently focusing upon an impact category rather than another one. In this regard it is worth making a consideration to pay due attention to comparing scenarios that are likely to be similar but which can, instead, show big differences just by expanding the investigated field.

## 4. Conclusions

In the framework of the environmental impact assessments, in the last years, LCA has emerged as a valuable decision-support tool for decision-makers, in both political and industrial areas. Consequently, LCA has assumed over the years a key role as a tool for identifying cradle-to-grave impacts of products in a multitude of sectors: among them, polymers is one of the most investigated, considering the uncountable number of disparate applications.

Through this literature review, the authors achieved their goal of reviewing and building upon LCAs in the Italian polymeric sector, to highlight the most relevant environmental issues.

It was found that LCA was used in the majority of those sectors where plastic products are utilized, namely:

- agriculture;
- food production and packaging;
- automotive;
- concrete-polymeric materials;
- geopolymers;
- polymeric materials in fuel cells.

In each sector and for each application, the Italian researchers proposed, on the basis of LCA results, sometimes from the cradle to the grave and at other times from the cradle to the gate, the best solution to minimize the environmental impact and ensure a viable choice also from an economic point of view. Food production and packaging resulted the most studied sector with LCA, but in all the other sectors we recorded increasing attention on the whole polymer’s life cycle and the need to show its sustainability.
